# COVID-19 transmission during swimming-related activities: a rapid systematic review

**DOI:** 10.1186/s12879-021-06802-4

**Published:** 2021-10-29

**Authors:** Sally Yaacoub, Joanne Khabsa, Rayane El-Khoury, Amena El-Harakeh, Tamara Lotfi, Zahra Saad, Zeina Itani, Assem M. Khamis, Ibrahim El Mikati, Carlos A. Cuello-Garcia, Francisca Verdugo-Paiva, Gabriel Rada, Holger J. Schünemann, Nesrine Rizk, Elie A. Akl

**Affiliations:** 1 Clinical Research Institute, American University of Beirut Medical Center, Beirut, Lebanon; 2 Health Research Methods, Evidence & Impact, McMaster University, Hamilton, Canada; 3 WHO Collaborating Center for Infectious Diseases, Research Methods and Recommendations; Michael G DeGroote Cochrane Canada Centre; GRADE Canada Centre, Hamilton, Canada; 4 Hull York Medical School, University of Hull, Hull, England; 5 Faculty of Medicine, American University of Beirut, Beirut, Lebanon; 6 Epistemonikos Foundation, Santiago, Chile; 7 UC Evidence Center, Cochrane Chile Associated Center, Pontificia Universidad Católica de Chile, Santiago, Chile; 8 Department of Internal Medicine, American University of Beirut, 11-0236 / CRI (E15), Beirut, 1107 2020 Lebanon

**Keywords:** COVID-19, Coronavirus, Swimming, Recreation, Social distancing, Personal protective equipment

## Abstract

**Background:**

There are uncertainties about mitigating strategies for swimming-related activities in the context of the COVID-19 pandemic. There is an opportunity to learn from the experience of previous re-openings to better plan the future one. Our objectives are to systematically review the evidence on (1) the association between engaging in swimming-related activities and COVID-19 transmission; and (2) the effects of strategies for preventing COVID-19 transmission during swimming-related activities.

**Methods:**

We conducted a rapid systematic review. We searched in the L·OVE (Living OVerview of Evidence) platform for COVID-19. The searches covered the period from the inception date of each database until April 19, 2021. We included non-randomized studies for the review on association of COVID-19 transmission and swimming-related activities. We included guidance documents reporting on the strategies for prevention of COVID-19 transmission during swimming-related activities. We also included studies on the efficacy and safety of the strategies. Teams of two reviewers independently assessed article eligibility. For the guidance documents, a single reviewer assessed the eligibility and a second reviewer verified the judgement. Teams of two reviewers extracted data independently. We summarized the findings of included studies narratively. We synthesized information from guidance documents according to the identified topics and subtopics, and presented them in tabular and narrative formats.

**Results:**

We identified three studies providing very low certainty evidence for the association between engaging in swimming-related activities and COVID-19 transmission. The analysis of 50 eligible guidance documents identified 11 topics: ensuring social distancing, ensuring personal hygiene, using personal protective equipment, eating and drinking, maintaining the pool, managing frequently touched surfaces, ventilation of indoor spaces, screening and management of sickness, delivering first aid, raising awareness, and vaccination. One study assessing the efficacy of strategies to prevent COVID-19 transmission did not find an association between compliance with precautionary restrictions and COVID-19 transmission.

**Conclusions:**

There are major gaps in the research evidence of relevance to swimming-related activities in the context of the COVID-19 pandemic. However, the synthesis of the identified strategies from guidance documents can inform public health management strategies for swimming-related activities, particularly in future re-opening plans.

**Supplementary Information:**

The online version contains supplementary material available at 10.1186/s12879-021-06802-4.

## Background

In January 2020, a new coronavirus was identified in China and has since spread worldwide, causing an outbreak. This novel coronavirus, named severe acute respiratory syndrome coronavirus 2 (SARS-CoV-2) emerged in the city of Wuhan in China. Coronavirus disease 2019 (COVID-19) is the acute respiratory disease caused by SARS-CoV-2 [1]. In March 2020, the WHO declared the novel coronavirus outbreak a pandemic. It has since triggered a global lockdown and resulted in an unprecedented recession. As of April 2021, it has already infected more than 150 million people and accounted for more than 3 million deaths worldwide [2].

In response to the growing pandemic, governments across the world used partial or full lockdowns on their populations as part of the public health efforts to flatten the epidemiologic curve and avoid a surge of cases that would overwhelm their healthcare systems [3, 4]. As part of those lockdowns, airports and educational establishments are closed, businesses, both public and private, are forced to adjust their operations, and most employees are asked to work from home [5].

Following each lockdown, governments implement re-opening plans with a major aim of safely emerging from economic recessions [6]. Those plans include policies and guidance for re-opening public places for outdoor activities, including swimming-related activities. It would be important to learn from the experience of those re-openings, to better plan the future re-opening, e.g., in terms of enhancing mitigating strategies for different activities.

Swimming-related activities are undeniably important for the physical and mental well-being of millions of individuals forced into confinement and isolation for prolonged periods. However, swimming in a pool or a lake, and sunbathing on the beach or shore, imply close proximity between individuals and high frequency of touching common surfaces. These factors would increase the risk of virus transmission. Indeed, SARS-CoV-2 is reported to spread through droplet and potentially airborne transmission [7]. Water-borne transmission is still not certain. A well-publicized event was a Fourth of July celebration in a Michigan lake, where individuals contracted COVID-19 [8].

The objective of this study was to systematically review the evidence on (1) the association between engaging in swimming-related activities and COVID-19 transmission; and (2) the effects of strategies for preventing COVID-19 transmission during swimming-related activities.

## Methods

We conducted a rapid systematic review to identify, select, abstract, assess, and synthesize the available evidence addressing our questions of interest. We report this rapid systematic review following the guidelines of the preferred reporting items for systematic reviews and meta-analyses (PRISMA) checklist. We registered the protocol in Open Science Framework (osf.io/38hrw). While we initially aimed to conduct a living systematic review (as stated in the protocol), we opted to conduct a regular (non-living) review due to feasibility issues.

### Eligibility criteria

For the review of association, the population of interest was the general public. The exposure of interest was engaging in any swimming-related activity, such as going to a pool, a beach, a river or a lake. The outcome of interest was COVID-19 infection. Eligible study designs consisted of non-randomized studies (including cohort studies, case control studies, case series and case reports). We did not have any language restrictions. We excluded environmental studies, mechanistic studies, modeling studies, reviews, letters to the editor, conference abstracts, commentaries and opinion pieces.

For the review of strategies, we included guidance documents reporting on the prevention of COVID-19 transmission during swimming-related activities. Eligible documents should have provided a substantial description of a strategy. We also included studies of any language on the efficacy and safety of these strategies. We excluded mechanistic studies. We excluded documents that did not address swimming-related activities (e.g., rehabilitation pools, fishing), were only regulatory (i.e., official documents stating the phases of opening), solely referred to other guidance documents, were press releases or were in the form of Frequently Asked Questions (FAQs).

### Search strategy

For both, the review of association and the review of strategies, we systematically searched in L·OVE (Living OVerview of Evidence) platform for COVID-19, a system that maps PICO questions to a repository developed by Epistemonikos Foundation. This repository is continuously updated through searches in 41 sources including electronic databases, preprint servers, trial registries and other resources relevant to COVID-19. The searches covered the period from the inception date of each database until April 19, 2021. The results of the searches in the individual sources were de-duplicated by an algorithm that compares unique identifiers (database ID, DOI, trial registry ID), and citation details (i.e., author names, journal, year of publication, volume, number, pages, article title, and article abstract). We ran a search about ‘swimming-related activities’, using the search methods of the COVID-19 L·OVE platform [9]. Additional file 1: Appendix 1 provides the list of terms and databases used in those searches. Additionally, we screened the references of included studies and aimed to screen relevant systematic reviews.

For the review of strategies, we also searched the websites of relevant guideline-producing organizations, initially up to June 10, 2020, for relevant guidance documents. We compiled a list of these organizations starting with a list we had developed for another project on guidance documents (unpublished). Also, we searched the International Association for Sports and Leisure Facilities (IAKS) website for news on ‘pools and aquatic facilities’ [10] to identify further organizations. In addition, we identified countries that were easing travel restrictions [11], and searched the websites of every state of the United States (U.S.). Then, we performed a general Google search as well as a Google search restricted to governmental websites (.gov). We used a combination of terms referring to COVID-19 and pools, beaches, rivers and lakes. We also screened the references of included guidance documents. We updated the list of guidance documents on April 19, 2021.

### Study selection

The information matching the search strategy was sent in real-time to the L·OVE platform where at least two reviewers independently screened the titles and abstracts yielded against the inclusion criteria. We obtained the full texts for all the studies that appeared to meet the inclusion criteria or required further analysis. Then, we judged the eligibility of these studies. We resolved any conflicts by discussion, or with the help of a third reviewer. We recorded the primary reason for exclusion at the full-text screening stage and we listed those studies with the reasons for exclusion.

For the identified guidance documents, the first reviewer assessed the eligibility and the second reviewer verified the judgement of the first reviewer. We resolved any conflicts by discussion, or with the help of a third reviewer.

### Data extraction and synthesis

Teams of two reviewers extracted data independently using a pilot-tested form. They resolved disagreements by consensus, or with the help of a third reviewer as needed. The data we extracted from studies of association included study population, setting, and data on risk of COVID-19 transmission. For the review of strategies, we extracted data on the entity that produced the guidance document, date of last update, country and language, setting of the guidance (i.e., beach and/or pool), and focus (either specifically addressing swimming activities, or addressing other activities). We abstracted from each document all relevant recommendations in detail and analyzed them to identify the topics and subtopics covered. In addition, we abstracted data from studies reporting on efficacy and safety of these strategies. Abstracted data included study population, setting, types of strategies, and data on COVID-19 transmission following the implementation of strategies. We performed the risk of bias assessment using ROBINS-I tool for non-randomized studies and planned to use the Cochrane risk of bias tool for randomized studies, when applicable.

We aimed to quantitatively synthesize data on association and data on the effects of strategies. However, this was not possible, and we therefore summarized the findings of included studies narratively. We synthesized information from guidance documents according to the identified topics and subtopics and presented them in tabular and narrative formats. Where applicable, we reported the percentage of guidance documents addressing the topics or subtopics. For topics with high variability or inconsistency, we made sure to present the spectrum of recommendations given. Otherwise, we attempted to represent agreement across guidance documents where applicable. We graded the certainty of the evidence using the GRADE approach [12].

## Results

### Review of association

**Fig. 1:**
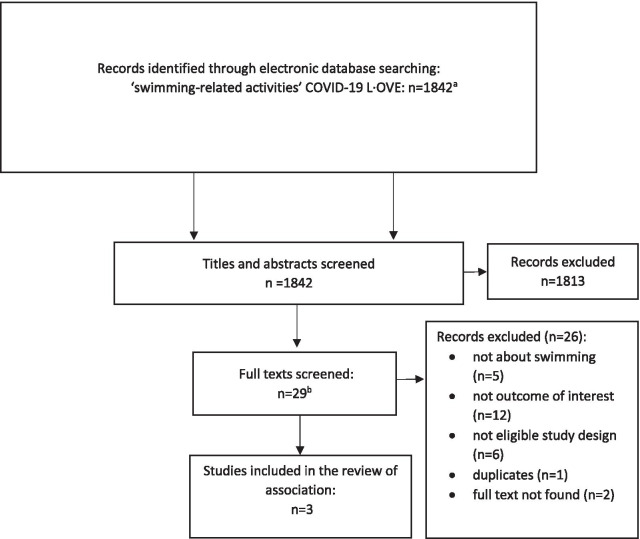
Study selection for the COVID-19 L·OVE platform search. ^a^Records are de-duplicated by an algorithm that compares unique identifiers (database ID, DOI, trial registry ID), and citation details (i.e. author names, journal, year of publication, volume, number, pages, article title, and article abstract). ^b^Three of these were guidance documents, two of which were included in the review of strategies

Figure 1 presents the results of the selection process conducted in the COVID-19 L·OVE platform. After deduplication and title and abstract screening, COVID-19 L·OVE platform identified 29 potentially eligible full text studies. We excluded 26 studies for the following reasons: not about swimming (n = 5), not outcome of interest (n = 12), not eligible study design (n = 6), duplicates (n = 1), full text not found (n = 2) (see Additional file 1: Appendix 2a for more details). This resulted in three eligible studies for the review of association [13–15].

Termansen et al., a preprint publication, conducted a retrospective questionnaire-based single arm cohort to describe the extent of transmission of SARS-CoV-2 at indoor swimming activities in Danish swimming clubs during August–December 2020. Data were collected from an official contact person from each swimming club using an electronic-based questionnaire. Out of a total of 162 risk episodes (i.e., where a SARS-CoV-2 positive subject was participating in a swimming activity), eight (4.9%) led to transmission to 23 participants. The transmission was reported to be 43.5 and 4.7 participants per 100,000 pool activity hours in competitive swimming and recreational swimming, respectively. None was reported for water polo as there was no transmission episode. The authors also noted that 23 swimmers from the same club were infected with SARS-CoV-2 at a training camp. However, other clubs training in the same swimming pool in the same period did not report infected participants. The authors hypothesized that transmission could have resulted from other activities during the training camp (e.g. sleeping in dormitories, dining and socializing together) [15].

Bao et al. conducted an epidemiological study to investigate an outbreak of COVID-19 infection. The index case had frequented an entertainment site, which contained a floor for public bathing. The patient took a public bath on 2 consecutive days. A total of 12 bath-related infections were attributed directly to that patient: ten among bathers, and two among workers at the site. One of these bathers subsequently infected 19 colleagues and family members at consecutive dinners, and one of the two workers infected 41 individuals, of which seven were bathers. Authors noted that the secondary attack rate at the pool was significantly lower than that outside the pool (i.e. colleagues and family clusters). They hypothesized that this could be due to the high temperature (between 18 and 42 °C) and humidity (60–80%) at the entertainment site, which ‘suppressed virus transmissibility’ [13].

Luo et al. reported data for nine confirmed COVID-19 patients who frequented the same ‘bath center’ and were hospitalized in the Jiangsu Province of China. The bath center contained a swimming pool, showers, and sauna. The authors reported that the first patient showered in the center, while the next seven patients showered, used the sauna, and swam in the pool, and the 9th case was among staff. The study concluded that transmissibility of COVID-19 “showed no signs of weakening in warm and humid conditions” (temperature was 25–41 °C and humidity was 60%) [14].

We judged the evidence supporting the conclusions of these three studies to be of very low certainty (one retrospective single arm study and two small case series with no adjustment for confounding).

### Review of strategies

We identified in the COVID-19 L·OVE one study assessing the efficacy but not safety of strategies for preventing COVID-19 transmission during swimming-related activities (study by Termansen et al. described above). The study found no association between implementation of restrictions and risk of SARS-CoV-2 transmission during indoor swimming activities. Restrictions included distancing, personal hygiene, limiting use of shared equipment, limiting physical activity around the pool area, and raising chlorine content. The authors reported that this analysis had low statistical power [15]. We judged the study as having serious risk of bias and providing very low certainty evidence.

We identified a total of 73 guidance documents (71 through hand-searching and two through COVID-19 L·OVE). We excluded 23 documents for the following reasons: regulatory type (n = 5), not about COVID-19 (n = 2), not about swimming-related activity or brief section on swimming (n = 5), press release (n = 3), information in the form of FAQs (n = 4), other (n = 4) (Additional file 1: Appendix 2b).

We included a total of 50 guidance issued by 50 organizations and published in 76 documents (15 guidance were published in more than one document) (Additional file 1: Appendix 3). Table 1 shows the characteristics of included guidance documents.

**Table 1:** Characteristics of included guidance documents (n = 50)

		n (%)
Type of organization	Governmental	40 (80)
	Non-governmental	10 (20)
Country	U.S	31 (62)
	European countries	12 (24)
	Other	7 (14)
Language	English	41 (82)
	Other	9 (18)
Specifically addresses swimming activities	Yes	41 (82)
	No (addresses other activities)	9 (18)
Setting^a^	Pool setting	47 (94)
	Beach setting	11 (22)

*U.S.* United States

^a^Percentages do not add to 100 due to overlap

The majority of those documents specifically addressed swimming activities (82%), were issued by governmental organizations (80%), were issued by U.S. based organizations (62%), were published in English (82%) and addressed pool (as opposed to beach) related activities (94%). The documents addressed 11 topics related to swimming activities (Table 2).

**Table 2:** Topics and subtopics addressed in included guidance documents (n = 50)

Topic (n, %)	Subtopic
Ensuring social distancing (50, 100%)	Capacity control
	Distancing measures
	Banning/restricting access to certain locations
	Banning/restricting access to certain activities
Ensuring personal hygiene (45, 90%)	Promoting personal hygiene practices
	Facilitating personal hygiene practices
	Ensuring adequate hygiene supplies
	Ensuring safe payment procedures
Using personal protective equipment (38, 76%)	–
Eating and drinking (25, 50%)	Food services
	Sharing food
	Social distancing
	Drinking
Maintaining the pool (33, 66%)	Disinfectants
	Maintenance parameters
	Filtration and overflow outlets
	Cleaning
	Quality control
Managing frequently touched surfaces (48, 96%)	Cleaning and disinfecting areas
	Frequency of cleaning and disinfection
	Handling of towels
	Additional control measures
Ventilating indoor spaces (21, 42%)	–
Screening and management of sickness (45, 90%)	Screening for COVID-19 symptoms and precautions
	Symptomatic and high risk individuals
	Policies and procedures
Delivering first aid (21, 42%)	Providing first aid
	Lifeguard duties
	Lifeguard PPE
	Lifeguard distancing and contact
Raising awareness (36, 72%)	Channels and content
	Location of posted signs
	Target audience considerations
Vaccination (3, 6%)	–

**Fig. 2:**
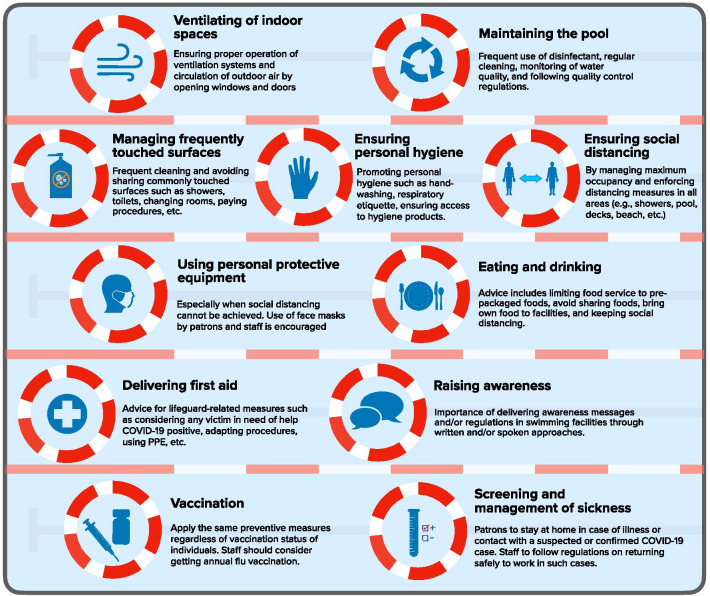
Strategies for preventing SARS-CoV-2 transmission during swimming-related activities

Additional file 1: Appendix 4 presents a detailed description of recommendations organized by topics and sub-topics. Figure 2 represents an infographic summarizing the recommendations by topic. We present a brief description of those recommendations in the subsequent sections for each of the 11 topics.

#### Ensuring social distancing

- Managing capacity: Most documents addressed limiting the capacity of facilities (n = 44, 88%). Documents defined limits using one of the following parameters: a percentage of usual capacity (n = 14, 32%) (typically 50% of usual capacity with values ranging between 25 and 75%); number of persons allowed per surface area (n = 8, 18%); gathering limits set by local authorities (n = 7, 16%), or a set maximum number (n = 4, 9%). To ensure adherence to capacity limits, 30 documents mentioned ways to limit number of guests at a given time (n = 30, 68%), such as a reservation system (n = 18, 60%); and 18 documents mentioned ways to limit number of users of specific facilities at a given time (n = 18, 41%), such as reducing the number available for use (n = 14, 78%).
- Distancing measures: Most documents advised on distancing measures (n = 49, 98%). The majority of documents recommended either 6 feet (n = 31, 63%) or 2 m (n = 4, 8%); while the rest recommended 1.5 m (n = 5, 10%) or 1 m (n = 2, 4%). In order to ensure adherence to the advised distancing measures, documents recommended the installation of physical barriers (n = 24, 49%) the use of visual cues (n = 30, 61%), changing the space layout (n = 38, 78%), and managing traffic flow of individuals (n = 20, 41%). Other measures included restricting access to specific areas (n = 24, 49%), restricting specific activities (n = 24, 49%), modifying the schedule to reduce patrons’ and/or staff’s interactions (n = 19, 39%), pool and area monitoring (n = 18, 37%), and swimming-specific distancing measures (n = 14, 29%), such as limiting swimming to a single person per lane (n = 4, 29%)

#### Ensuring personal hygiene

A total of 45 documents addressed ensuring personal hygiene (n = 45, 90%). Most of these documents mentioned promoting personal hygiene practices (n = 35, 78%), such as handwashing (n = 33, 94%), respiratory etiquette (n = 17, 49%) and avoiding touching the face with unwashed hands (n = 11, 31%). Other measures included facilitating personal hygiene practices (n = 27, 60%) (e.g. by ensuring access to hand sanitizer dispensers and/or washing stations), ensuring adequate hygiene supplies (n = 33, 73%), and practicing safe payment procedures (n = 19, 42%).

#### Using personal protective equipment (PPE)

The majority of guidance documents (n = 38, 76%) mentioned the use of PPE, especially when physical distancing cannot be achieved (n = 16, 42%). More specifically, documents mentioned the wear of face coverings (masks or cloth face coverings) by patrons (n = 27, 71%), as well as by staff (n = 26, 68%) and lifeguards (n = 7, 18%). There was a general agreement that masks should not be worn inside the water and damp areas (n = 23, 61%). Other PPE options included glove use by staff (n = 9, 24%) when cleaning or when handling towels.

#### Eating and drinking

Twenty-five documents addressed eating (n = 25, 50%). Five guidance documents mentioned limiting services to prepackaged food (n = 5, 20%), seven mentioned discouraging sharing food among patrons or employees (n = 7, 28%), and eleven mentioned approaches to ensure social distancing in eating areas (n = 11, 44%). Twelve documents tackled management procedures for drinking (n = 12, 48%). Of these, six documents suggested keeping the drinking fountains functional (n = 6, 50%), five documents suggested suspending or restricting their use (n = 5, 42%) and four documents encouraged visitors to bring their own water/fluids (n = 4, 33%).

#### Maintaining the pool

Out of the 33 guidance documents that reported on pool maintenance, 22 reported on the use of a disinfectant for the pool (n = 22, 67%). The most reported disinfectant was chlorine (n = 20, 91%), followed by bromine (n = 9, 41%). Ten documents reported on measures for cleaning the pool (n = 10, 30%), including proper stocking of products (n = 1, 10%). As part of quality control (n = 23, 70%), 14 guidance documents suggested monitoring (n = 14, 61%) through monitoring the pool chemistry (n = 8, 57%) and keeping monitoring records (n = 3, 21%), alongside other measures. An additional measure of quality control is ensuring compliance with rules and regulations (n = 18, 78%), whether state or local regulations (n = 13, 72%), CDC considerations (n = 3, 17%), WHO strategies (n = 1, 6%), licensing conditions (n = 1, 6%), or manufacturers’ guidelines (n = 1, 6%).

#### Managing frequently touched surfaces

The majority of documents addressed managing frequently touched surfaces (n = 48, 96%). Frequently touched surfaces included: toilets, restrooms and dressing rooms (n = 36, 75%); the pool and beach area, including deck and equipment (n = 28, 58%); and other common areas/surfaces, including waiting rooms, door knobs, pool ladders and/or lifts (n = 34, 71%). The recommended frequency of cleaning was highly variable across documents (n = 33, 69%). Control measures to decrease the frequency of touching surfaces included the prohibition of sharing objects (n = 19, 40%), restricting the use and access to facilities for which disinfection between different users is not practical (n = 12, 25%), providing no-touch or foot pedal installations (n = 9, 19%), and using contactless payment methods or online transactions (n = 16, 33%). Four guidance documents mentioned requiring guests to provide their own towels (n = 4, 8%), and seven mentioned measures for appropriate washing and drying of towels if provided (n = 7, 15%).

#### Ventilation of indoor spaces

Twenty-one documents addressed ventilation in indoor spaces (n = 21, 42%). Recommendations included ensuring that ventilation systems operate properly (n = 10, 48%) and increasing circulation of outdoor air by opening windows and doors or by other methods (n = 16, 76%).

#### Screening and management of sickness

A total of 45 documents reported on screening and management of suspected or confirmed COVID-19 cases (n = 45, 90%). From those, 34 documents highlighted the need for patrons or staff to stay at home in case of illness or contact with a suspected or confirmed COVID-19 case (n = 34, 76%). For employees, the time duration after which returning to work was allowed varied substantially across documents (n = 8, 18%). In addition, specific measures were highlighted in seventeen documents to protect patrons or staff that are considered high risk individuals (n = 17, 38%), such as allocating access time for this group (n = 4, 24%), taking extra precautions (n = 3, 18%), and refraining from going to the pool (n = 6, 35%).

#### Delivering first aid

A total of 21 documents mentioned first aid and/or lifeguard-related measures (n = 21, 43%). Ten mentioned measures relating to the provision of first aid (n = 10, 48%), such as treating any victim as COVID-19 positive until otherwise determined (n = 2, 20%), provision of appropriate cardiopulmonary resuscitation (CPR) equipment (e.g. one-way valve masks for CPR; n = 6, 60%), and revising CPR protocol (n = 4, 40%). Ten documents (n = 10, 48%) highlighted that lifeguards were expected not to perform duties that distract them from the responsibilities of lifeguarding (including monitoring hand washing, mask wearing, and social distancing). Ten documents mentioned that lifeguards should wear PPE (n = 10, 48%), and eight documents mentioned that lifeguards should limit contact and abide by social distancing (8, 38%).

#### Raising awareness

Different documents addressed the importance of delivering awareness messages and/or regulations in swimming facilities through written (posting) and/or oral (broadcasting) approaches (n = 36, 72%). Posted signs and/or broadcasts mostly covered measures on social distancing (n = 26, 72%), hand hygiene (n = 16, 44%), and measures taken in case of symptomatic individuals (n = 21, 58%), such as not being allowed to enter. Signs were advised to be posted at multiple locations (n = 18, 50%), mostly at the entrance of facilities. Signs needed to be tailored to the target audience, including being in understandable language (n = 2, 6%).

#### Vaccination

Three documents reported on vaccination-related guidance (n = 3, 6%). Two documents advised that facilities apply the same preventive measures regardless of vaccination status of individuals (n = 2, 67%). The third document reported that staff should consider getting an annual flu vaccination (n = 1, 33%).

## Discussion

### Summary of findings

In summary, we identified three studies providing very low certainty evidence for the association between engaging in swimming-related activities and COVID-19 transmission. We also identified 50 guidance documents on strategies to prevent COVID-19 transmission during swimming-related activities. The guidance documents addressed the following 11 topics: pool maintenance, social distancing, personal protective equipment (mostly face coverings), managing frequently touched surfaces, eating and/or drinking, first aid, personal hygiene, screening and management of sickness, raising awareness, and vaccination. We identified one study assessing the efficacy of strategies to prevent COVID-19 transmission, showing no association between compliance with precautionary restrictions and transmission. It provided very low certainty evidence.

### Strengths and limitations

This rapid systematic review has a number of strengths. First, we used a very extensive and thorough search strategy across all languages. Through using the COVID-19 L·OVE platform, we were able to utilize artificial intelligence and machine learning algorithms to search, deduplicate search results, and identify potentially eligible studies in real time (given the continuous update of the platform). A team of diverse experienced reviewers finalized the selection, data abstraction and synthesis steps. Also, we searched for guidance documents through checking the websites of all states in the U.S., of countries easing travel restrictions and of associations concerned with sports and leisure activities. Although we conducted a rapid review, we followed standard methods of conducting systematic reviews to a large extent [16, 17]. One limitation of this study is that we did not include mechanistic or environmental studies. Another limitation is that the search for the guidance documents was not systematic. A major limitation is that the included guidance documents were not formally developed guidelines (i.e., would not meet the criteria of trustworthy guidelines, or score well on the AGREE II tool). Another limitation is that some of the guidance documents focused on a specific phase of reopening, and could have been later on modified, e.g., to address another phase of the reopening. However, our summarized strategies reflect the most recent guidance available at the time of our data retrieval.

### Interpretation of findings

Our findings show a major gap in the research evidence on the association between engaging in swimming-related activities and COVID-19 transmission, and on the efficacy and safety of strategies related to this topic. This is consistent with research on transmission through other activities including singing and playing wind instruments [18].

To a large extent, the documents were consistent particularly in relation to the broad principles of social distancing, use of PPE, personal hygiene, and cleaning surfaces. Documents also commonly highlighted measures addressing individuals considered high risk (e.g. older adults). However, there were some variability, and even some inconsistencies in how exactly to apply those principles. For example, there was some variability in how to implement ‘capacity control’ as part of ‘ensuring social distancing’. Guidance documents proposed one or more of a number of ways of defining maximum capacity, e.g. as a percentage, persons per square meter of surface area, maximum fixed number of persons, or other. Areas with clear inconsistences were opening versus closing food services; keeping drinking fountains functional versus suspending their use; allowing versus banning specific activities (e.g. swimming classes), and lifeguard duties (whether or not they include enforcing social distancing).

### Implications for public health practice

In light of the scarce evidence on the association of COVID-19 transmission with swimming-related activities, or on the effectiveness of mitigating interventions, the synthesis of the identified strategies from guidance documents can inform public health management strategies for swimming-related activities, particularly in future re-opening plans. In addition, pool managers and operators should take into account the relevance, acceptability, feasibility, availability of resources, and prevalence of COVID-19, upon implementing COVID-19 prevention strategies.

### Implications for research

There is a need for evidence on the association of COVID-19 transmission and engaging in swimming-related activities and on the efficacy and safety of the proposed strategies. Although we found scarce evidence for swimming-related activities, we are aware of the availability of streams of evidence from other contexts (e.g. mode of transmission in healthcare setting or community) and from other types of studies (e.g., mechanistic and modeling studies) that could be relevant to our topic. One can build on those streams of evidence to support an analytical framework addressing the different activities and exposures, mode of transmission, mitigating factors and modifying factors for COVID-19 in swimming-related activities.

## Supplementary Information

**Additional file 1: Appendix 1a.** COVID-19 L·OVE search terms. **Appendix 1b.** COVID-19 L·OVE list of searched databases. **Appendix 2a.** Excluded studies (n = 26). **Appendix 2b.** Excluded guidance documents (n = 23). **Appendix 3.** Characteristics of included guidance documents (n = 50). **Appendix 4.** Detailed description of recommendations organized by topics and sub-topics (n = 50).

## Data Availability

The datasets generated and/or analysed during the current study are not publicly available, but are available from the corresponding author on reasonable request.

## Abbreviations

COVID-19: Coronavirus disease 2019
SARS-CoV-2: Severe acute respiratory syndrome coronavirus 2
PPE: Personal protective equipment
L·OVE: Living OVerview of Evidence
PRISMA: Preferred reporting items for systematic reviews and meta-analyses
FAQ: Frequently Asked Question
IAKS: International Association for Sports and Leisure Facilities
U.S.: United States
CPR: Cardiopulmonary resuscitation
