# Supplementary material for: COVID-19 transmission during swimming-related activities: a rapid systematic review
Source: BMC Infect Dis. 2021 Oct 29;21:1112. doi: 10.1186/s12879-021-06802-4 (PMC8553516; doi:10.1186/s12879-021-06802-4)
Supplement: Supplementary file 1 — Additional file 1: Appendix 1a. COVID-19 L·OVE search terms. Appendix 1b. COVID-19 L·OVE list of searched databases. Appendix 2a. Excluded studies (n = 26). Appendix 2b. Excluded guidance documents (n = 23). Appendix 3. Characteristics of included guidance documents (n = 50). Appendix 4. Detailed description of recommendations organized by topics and sub-topics (n = 50). [file 12879_2021_6802_MOESM1_ESM.docx]

**COVID-19 transmission during swimming-related activities: a rapid systematic review**

**Authors and authors’ details:**

| **Author Name** | **Degree** | **Affiliation(s) (include city and country)** | **Email** | **Conflicts of interest** |
| --- | --- | --- | --- | --- |
| Sally Yaacoub | BSPharm, MPH | Clinical Research Institute, American University of Beirut Medical Center, Beirut, Lebanon | Sally.yaacoub@gmail.com | None |
| Joanne Khabsa | BSPharm, MPH | Clinical Research Institute, American University of Beirut Medical Center, Beirut, Lebanon | jk81@aub.edu.lb | None |
| Rayane El-Khoury | MPH | Clinical Research Institute, American University of Beirut Medical Center, Beirut, Lebanon | re130@aub.edu.lb | None |
| Amena El-Harakeh | MPH | Clinical Research Institute, American University of Beirut Medical Center, Beirut, Lebanon | amenaharakeh@gmail.com | None |
| Tamara Lotfi | MD, MPH | Department of Health Research Methods, Evidence & Impact, McMaster University, Hamilton, Canada | lotfit@mcmaster.ca | None |
| Zahra Saad | MS | Clinical Research Institute, American University of Beirut Medical Center, Beirut, Lebanon | zs53@aub.edu.lb | None |
| Zeina Itani | MPH | Clinical Research Institute, American University of Beirut Medical Center, Beirut, Lebanon | zmi03@mail.aub.edu | None |
| Assem M. Khamis | MD, MPH | Hull York Medical School, University of Hull, Hull, England | amk88@mail.aub.edu | None |
| Ibrahim El Mikati | MD | Faculty of Medicine, American University of Beirut | ike05@mail.aub.edu | None |
| Carlos A. Cuello Garcia | MD, PhD | Health Research Methods Evidence and Impact, McMaster University | cuelloca@mcmaster.ca | None |
| Francisca Verdugo | DDS, MSc | Epistemonikos Foundation, Santiago, Chile  UC Evidence Center, Cochrane Chile Associated Center, Pontificia Universidad Católica de Chile, Santiago, Chile | fverdugo@epistemonikos.org | None |
| Gabriel Rada | MD | Epistemonikos Foundation, Santiago, Chile  UC Evidence Center, Cochrane Chile Associated Center, Pontificia Universidad Católica de Chile, Santiago, Chile | radagabriel@epistemonikos.org | None |
| Holger J. Schünemann | MD, PhD | Health Research Methods, Evidence & Impact; Medicine; WHO Collaborating  Center for Infectious Diseases, Research Methods and Recommendations; Michael G  DeGroote Cochrane Canada Centre; GRADE Canada Centre | holger.schunemann@mcmaster.ca | None |
| Nesrine Rizk | MD | Department of Internal Medicine, American University of Beirut, Beirut, Lebanon | nr00@aub.edu.lb | None |
| Elie A. Akl | MD, MPH, PhD | Department of Internal Medicine, American University of Beirut, Beirut, Lebanon | ea32@aub.edu.lb | None |

**Corresponding author:**

Elie A. Akl, MD, MPH, PhD

Department of Internal Medicine

American University of Beirut

P.O.Box 11-0236 / CRI (E15)

Riad-El-Solh Beirut 1107 2020

Beirut – Lebanon

T: + 961 1 374374

Email: [ea32@aub.edu.lb](mailto:ea32@aub.edu.lb)

**Additional file 1.**

Table of Contents

[**Appendix 1a:** COVID-19 L·OVE search terms 5](#_Toc71057741)

[**Appendix 1b:** COVID-19 L·OVE list of searched databases 6](#_Toc71057742)

[**Appendix 2a:** Excluded studies (N=26) 7](#_Toc71057743)

[**Appendix 2b:** Excluded guidance documents (N=23) 8](#_Toc71057744)

[**Appendix 3:** Characteristics of included guidance documents (N=50) 12](#_Toc71057745)

[**Appendix 4:** Detailed description of recommendations organized by topics and sub-topics (N=50) 34](#_Toc71057746)

[**1.** **Ensuring social distancing (n=50)** 34](#_Toc71057747)

[**2.** **Ensuring personal hygiene (n=45)** 43](#_Toc71057748)

[**3.** **Using personal protective equipment (PPE) (n=38)** 45](#_Toc71057749)

[**4.** **Eating and drinking (n=25)** 47](#_Toc71057750)

[**5.** **Maintaining the pool (n=33)** 49](#_Toc71057751)

[**6.** **Managing frequently touched surfaces (n=48)** 51](#_Toc71057752)

[**7.** **Ventilation of indoor spaces (n=21)** 54](#_Toc71057753)

[**8.** **Screening and management of sickness (n=45)** 55](#_Toc71057754)

[**9.** **Delivering first aid (n=21)** 58](#_Toc71057755)

[**10.** **Raising awareness (n=36)** 60](#_Toc71057756)

[**11.** **Vaccination (n=3)** 62](#_Toc71057757)

# **Appendix 1a:** COVID-19 L·OVE search terms

1. **General search: COVID-19 (SARS-CoV-2 infection affecting humans):**

coronavir* OR coronovirus* OR betacoronavir* OR "beta-coronavirus" OR "beta-coronaviruses" OR "corona virus" OR "virus corona" OR "corono virus" OR "virus corono" OR hcov* OR covid* OR "2019-ncov" OR cv19* OR "cv-19" OR "cv 19" OR "n-cov" OR ncov* OR (wuhan* AND (virus OR viruses OR viral)) OR "2019-ncov-related" OR "cv-19-related" OR "n-cov-related" OR sars* OR sari OR "severe acute respiratory syndrome" OR antisars* OR "anti-sars-cov-2" OR "anti-sars-cov2" OR "anti-sarscov-2" OR "anti-sarscov-2" OR "post-COVID-19" OR "Not-of-COVID-19" OR "corona patients" OR "article-covid-19" OR "post-covid-19" OR "post-covid" OR "with-covid-19" OR "pre-covid" OR "pre-covid-19" OR "with-covid" OR "anti-covid-19" OR "n-covid" OR "no-covid"

1. **Specific search for the filter ‘swimming-related activities’**

(pools OR bath* OR swim* OR swam* OR paddl* OR beach* OR lake* OR river* OR ocean* OR sea OR shore* OR sand OR (water* AND (sport* OR activities*)))

# **Appendix 1b:** COVID-19 L·OVE list of searched databases

1. Pubmed/medline (updated several times a day)
2. EMBASE (updated weekly)
3. CINAHL (updated weekly)
4. PsycINFO (updated weekly)
5. LILACS (Latin American & Caribbean Health Sciences Literature) (updated weekly)
6. Wanfang Database (updated every 2 weeks)
7. CBM - Chinese Biomedical Literature Database (updated every 2 weeks)
8. CNKI - Chinese National Knowledge Infrastructure (updated every 2 weeks)
9. VIP - Chinese Scientific Journal Database (updated every 2 weeks)
10. IRIS (WHO Institutional Repository for Information Sharing) (updated weekly)
11. IRIS PAHO (PAHO Institutional Repository for Information Sharing)) (updated weekly)
12. IBECS - Índice Bibliográfico Español en Ciencias de la Salud (Spanish Bibliographic Index on Health Sciences) (updated weekly)
13. Microsoft Academic (last searched: Sept 4, 2020)
14. ICTRP Search Portal (updated daily)
15. Clinicaltrials.gov (updated daily)
16. ISRCTN registry (updated daily)
17. Chinese Clinical Trial Registry (updated daily)
18. IRCT - Iranian Registry of Clinical Trials (updated daily)
19. EU Clinical Trials Register: Clinical trials for covid-19 (updated daily)
20. NIPH Clinical Trials Search (Japan) - Japan Primary Registries Network (JPRN) (JapicCTI, JMACCT CTR, jRCT, UMIN CTR) (updated daily, via ICTRP search portal)
21. UMIN-CTR - UMIN Clinical Trials Registry (updated daily, via ICTRP search portal)
22. JRCT - Japan Registry of Clinical Trials (updated daily, via ICTRP search portal)
23. JAPIC Clinical Trials Information (updated daily, via ICTRP search portal)
24. Clinical Research Information Service (CRiS), Republic of Korea (updated daily, via ICTRP search portal)
25. ANZCTR - Australian New Zealand Clinical Trials Registry (updated daily, via ICTRP search portal)
26. ReBec - Brazilian Clinical Trials Registry (updated daily, via ICTRP search portal)
27. CTRI - Clinical Trials Registry - India (updated daily, via ICTRP search portal)
28. RPCEC - Cuban Public Registry of Clinical Trials (updated daily, via ICTRP search portal)
29. DRKS - German Clinical Trials Register (updated daily, via ICTRP search portal)
30. LBCTR - Lebanese Clinical Trials Registry (updated daily, via ICTRP search portal)
31. TCTR - Thai Clinical Trials Registry (updated daily, via ICTRP search portal)
32. NTR - The Netherlands National Trial Register (updated daily, via ICTRP search portal)
33. PACTR - Pan African Clinical Trial Registry (updated daily, via ICTRP search portal)
34. REPEC - Peruvian Clinical Trial Registry (updated daily, via ICTRP search portal)
35. SLCTR - Sri Lanka Clinical Trials Registry (updated daily, via ICTRP search portal)
36. medRxiv (updated several times a day)
37. bioRxiv (updated several times a day)
38. SSRN Preprints (updated several times a day)
39. ChinaXiv (updated every 2 weeks)
40. SciELO Preprints (updated weekly)
41. Research Square (updated daily)

# **Appendix 2a:** Excluded studies (N=26)^^[[1]](#footnote-1)^^

| **Author, year** | **Reason for exclusion** |
| --- | --- |
| Barcala-Furelos R et al, 2020(1) | Not outcome of interest |
| Brian F, 2020(2) | Not eligible study design |
| Chowell G et al, 2021(3) | Not about swimming |
| Clark M, 2021(4) | Not outcome of interest |
| Costa RMR and Vigário PDS., 2020(5) | Not outcome of interest |
| da Silva Garbuioa MEM and Ribeirob EAW, 2020(6) | Not outcome of interest |
| Efstratiou MA and Tzoraki O, 2021(7) | Not outcome of interest |
| Heath S, 2020(8) | Not about swimming |
| Holland J, 2020(9) | Not about swimming |
| Jeong GS, 2020(10) | Not outcome of interest |
| Kane B et al, 2021(11) | Not outcome of interest |
| Liikkanen LA and Laukkanen JA, 2021(12) | Not outcome of interest |
| Madeline F, 2020(13) | Not eligible study design |
| Montagna MT et al, 2021(14) | Not outcome of interest |
| Pereira LCC. et al, 2021(15) | Not outcome of interest |
| Quigley AL et al, 2021(16) | Not about swimming |
| Ramya G et al, 2021 (17) | Not eligible study design^[[2]](#footnote-2)^ |
| Romano SV et al, 2020 (18) | Not eligible study design^2^ |
| Romano-Bertrand S et al, 2020(19) | Not eligible study design^2^ |
| Sarah L and Bruce M, 2020(20) | Not outcome of interest |
| Vandana R, 2020(21) | Not eligible study design |
| Wroclaw Medical University, 2020(22) | Not outcome of interest |
| Yang B et al, 2020(23) | Not about swimming |
| Yoel, 2021(24) | Full text not found |
| (No authors listed), 2020(25) | Full text not found |

# **Appendix 2b:** Excluded guidance documents (N=23)

| **Publishing organization or website** | **Name of document or web page** | **Link of document or web page** | **Reason for exclusion** |
| --- | --- | --- | --- |
| New Jersey Deparment of Health, Public Health & Food Protection Program | Public Recreational Bathing Frequently Asked Questions | <https://pooloperationmanagement.com/wp-content/uploads/2020/03/prb-faq-march-27-2020.pdf> | FAQ |
| Bureau of Environmental Health, Water Programs, Florida Department of Health | Voluntary Pool Closures | <http://www.floridahealth.gov/environmental-health/eh-preparedness/_documents/covid19-bp-voluntary-pool-closures.pdf> | Guidance for closing a pool |
| Ministry of Health, Government of Peru | Minsa dispone el regreso progresivo de la actividad física y recreacional | <https://www.gob.pe/institucion/minsa/noticias/179015-minsa-dispone-el-regreso-progresivo-de-la-actividad-fisica-y-recreacional> | Press release |
| Alabama (City of Gulf Shores) | Gulf Shores Reopens City Beaches and Facilities in Response to Safer at Home Order | <https://www.gulfshoresal.gov/DocumentCenter/View/9547/4-29-20-COVID-19-Update---Beaches-Open> | Press release |
| Division of Aquatic Resources, State of Hawaiʻi | Fishing Guidelines During COVID-19 | <https://dlnr.hawaii.gov/dar/announcements/fishing-guidelines-during-covid-19/> | Not about swimming-related activity |
| Kansas Office of the Governor | Ad Astra: A Plan to Reopen Kansas | <https://covid.ks.gov/wp-content/uploads/2020/05/Reopen-Kansas-Framework-v6.2-5.19.20.pdf> | Regulatory (public health order/phased opening) |
| Louisiana Department of Education (LDOE), State of Louisiana | Reopening Guidance for K-12 Summer Activities | <https://www.louisianabelieves.com/docs/default-source/covid-19-resources/k-12-supportive-guidance-for-summer-2020.pdf?sfvrsn=eb90981f_10> | Brief section on swimming |
| Department of Environment and Energy, Nebraska | Memorandum to Nebraska Pool Owners and Operators | <https://bloximages.newyork1.vip.townnews.com/omaha.com/content/tncms/assets/v3/editorial/4/df/4dfab02b-5780-56d6-b593-c3a825962aa3/5ea1acb28c1d0.pdf.pdf> | Regulatory (public health order/phased opening) |
| Department of Environmental Services, The State of New Hampshire | NHDES Watershed Management Bureau (WMB) COVID-19 FAQs & Information for the General Public | <https://www.des.nh.gov/covid19/documents/wmb-covid-faq.pdf> | FAQ |
| New Mexico Environment Department | Pool/Aquatics Program | <https://www.env.nm.gov/pools/pools-program/> | Refers to other organizations' guidance |
| Oregon Health Authority | Reopening Guidance | <https://govsite-assets.s3.amazonaws.com/r5A0l0n7TWnunBkBzwjV_le2342E.pdf> | Brief section on swimming |
| Pennsylvania Department of Environmental Protection | Swimming Pool Guidelines | <https://www.egovlink.com/public_documents300/plymouthtownship/published_documents/Code%20Enforcement/PA_DEP_SWIMMING_POOL_GUIDELINES.pdf> | Not about COVID-19 |
| South Dakota Department of Health (SD-DOH) | Coronavirus updates and information | <https://doh.sd.gov/news/Coronavirus.aspx> | Refers to other organizations' guidance |
| Health Department, Salt Lake Country | In and For Salt Lake County, State of Utah: Public Health Order In the matter of COVID-19 Pandemic (Order No.: 2020-08; May 29, 2020) | <https://slco.org/globalassets/1-site-files/health/programs/covid/pho/pho8.pdf> | Regulatory (public health order/phased opening) |
| Commonwealth of Virginia | Forward Virginia Guidelines | <https://www.virginia.gov/coronavirus/forwardvirginia/> | Regulatory (public health order/phased opening) |
| Division of Food and Recreational Safety, Department of Agriculture, Trade and Consumer Protection, Wisconsin | Guidance for Pool Operators, Water Parks During COVID-19 Pandemic | <https://datcp.wi.gov/Documents/WaterParksPoolsCovid19Tips.pdf> | Regulatory (public health order/phased opening) |
| Danish Police | Police measures against COVID-19 in Denmark | <https://politi.dk/en/coronavirus-in-denmark/extension-of-measures-during-the-covid19-outbreak-in-denmark> | FAQ |
| Ministry of Health, Cyprus | Coronavirus: Swimming pools open as Cyprus enters third phase of relaxation measures (Updated) | <https://cyprus-mail.com/2020/06/05/coronavirus-swimming-pools-open-as-cyprus-enters-third-phase-of-relaxation-measures/> | Press release |
| Kannewischer Collection | Protection plan for the re-opening of the thermal spas of the Kannewischer Collection | <https://iaks.sport/sites/default/files/downloads/Best%20practices/2020-05/Protection%20Plan%20Kannewischer%20Collection.pdf> | Commercial organization |
| Australian Institute of Sport | The Australian Institute of Sport framework for rebooting sport in a COVID-19 environment | <https://www.ais.gov.au/__data/assets/pdf_file/0008/730376/35845_AIS-Framework-for-rebooting-sport_FA.pdf> | Brief section on swimming |
| German Swimming Pool Association (DGfdB) | DGfdB technical report: Pandemic plan for baths [DGfdB Fachbericht: Pandemieplan Bäder] | <https://www.baederportal.com/fileadmin/user_upload/News/DGfdB-Pandemieplan-Baeder-Stand_2_Juni_2020.pdf> | Not about COVID-19 (pandemic plan in general, brief mention of COVID19) |
| Health Service Executive (HSE), Ireland | EHS Water O.U. Advice note to EHS on COVID-19 in chlorinated drinking water supplies and chlorinated swimming pools | <http://edepositireland.ie/bitstream/handle/2262/92275/Advice%20note%20to%20EHS%20on%20Coronavirus%20and%20Drinking%20%20Water%20and%20Swimming%20Pools_V3%205-3-2020.pdf?sequence=1&isAllowed=y> | FAQ |
| Sara Romano-Bertrand, Ludwig-Serge Aho Glele, Bruno Grandbastien, Didier Lepelletier, on behalf of the French Society for Hospital Hygiene | Preventing SARS-CoV-2 transmission in rehabilitation pools and therapeutic water environments | <https://www.sciencedirect.com/science/article/pii/S0195670120302887> | Not about swimming-related activity |

Abbreviations: FAQs, Frequently Asked Questions

# **Appendix 3:** Characteristics of included guidance documents (N=50)

| **ID** | **Publishing organization** | **Title of document(s)** | **Date of last update** | **Links of documents** | **Country** | **Language of document** | **Setting of document** | **Document specifically addresses swimming activities** |
| --- | --- | --- | --- | --- | --- | --- | --- | --- |
| 1 | Centers for Disease Control and Prevention (CDC) | 1- Considerations for public pools, hot tubs, and water playgrounds during COVID-19 | February 2021 | <https://www.cdc.gov/coronavirus/2019-ncov/community/parks-rec/aquatic-venues.html> | U.S. | English | Pool (public pools, hot tubs, and water playgrounds) | Yes |
|  |  | 2- Guidance for administrators in parks and recreational facilities | January 2021 | <https://www.cdc.gov/coronavirus/2019-ncov/community/parks-rec/park-administrators.html> |  |  |  |  |
| 2 | Spanish National Research Council (CSIC) | Informe sobre transmisión del SARS-CoV-2 en playas y piscinas [Report on SARS-CoV-2 transmission in beaches and pools] | May 2020 | <https://www.idaea.csic.es/wp-content/uploads/2020/05/INFORME_PlayasyPiscinas_.pdf> | Spain | Spanish | Beach and pool (sand from the beaches and banks, rivers and lakes, open pools, indoor pools, spas, steam baths and saunas) | Yes |
| 3 | Indiana State Department of Health | 1- Temporary closures of pools, spas, and other aquatic venues: Prevention of biofilm and Legionellae establishment | April 2020 | <https://www.in.gov/isdh/files/POOLStartup_COVID-19%202020415.pdf> | U.S. | English | Pool (pools, spas, and other aquatic venues) | Yes |
|  |  | 2- COVID-19 response recommendations for pools and aquatic facilities | November 2020 | <https://www.in.gov/isdh/files/COVID-19%20Pool%20Operation%20under%20EO%2020-48.pdf> |  |  |  |  |
|  |  | 3- COVID-19 Response Guidance for Pools and Aquatic Facilities | March 2021 | <https://www.in.gov/isdh/files/COVID-19%20Pool%20Operation%202021-03-16.pdf> |  |  |  |  |
| 4 | Istituto Nazionale per l'Assicurazione contro gli Infortuni sul Lavoro (Inail) and Istituto Superiore di Sanità (ISS) | Documento tecnico sull’analisi di rischio e le misure di contenimento del contagio da SARS-CoV-2 nelle attività ricreative di balneazione e in spiaggia | May 2020 | <https://media2-col.corriereobjects.it/pdf/2020/interni/Documento_tecnico_balneazione.pdf> | Italy | Italian | Beach and pool | Yes |
| 5 | Executive Office of Energy and Environmental Affairs, Massachusetts | 1- Boating and various other marine and inland waterway related activities – Phase IV, Step 1 | March 2021 | <https://www.mass.gov/doc/recreational-boating-and-various-other-marine-and-inland-waterway-related-activities-phase-iv-step-1-effective-32221/download> | U.S. | English | Beach and pool | Yes |
|  |  | 2- Beaches guidelines for managers | May 2020 | <https://www.mass.gov/doc/coastal-and-inland-beach-guidance-phase-i/download> |  |  |  |  |
|  |  | 3-Safety standards for coastal and inland beaches – Phase IV, Step 1 | March 2021 | <https://www.mass.gov/doc/safety-standards-for-coastal-and-inland-beaches-phase-iv-step-1-effective-32221/download> |  |  |  |  |
|  |  | 4-Safety standards for public and semi-public swimming pools – Phase IV, Step 1 | April 2021 | <https://www.mass.gov/doc/safety-standards-for-public-and-semi-public-swimming-pools-phase-iv-step-1-effective-32221/download> |  |  |  |  |
|  |  | 5- Outdoor recreation advisory task force | Not specified | <https://www.mass.gov/doc/outdoor-recreation-task-force-presentation-5-18-20/download> |  |  |  |  |
| 6 | County of San Luis Obispo Public Health Department | 1- Interim social/physical distancing and sanitation guidelines for shared residential swimming pools | Not specified | <https://www.slocounty.ca.gov/Departments/Health-Agency/Public-Health/Environmental-Health/Forms-Documents/Reference-Materials/Pools-and-Spas-Program-Reference-Documents/Interim-Requirements-for-Residential-Public-Swimmi.aspx> | U.S. | English | Pool | Yes |
|  |  | 2- Public swimming pools \| Summary of best practices during the COVID-19 pandemic | Not specified | <https://www.emergencyslo.org/en/resourcesGeneral/Public-Swimming-Pool-InfoGraphic-6-8-rev.pdf> |  |  |  |  |
|  |  | 3- Supplemental document 1 to the steps and reopen together (start) guide section 11. Public swimming pools, public spa pools. | May 2020 | <https://www.emergencyslo.org/en/resourcesGeneral/Public-Pools-Guidance.pdf> |  |  |  |  |
| 7 | Division of Public Health- Delaware Health and Social Services (DHSS) | COVID-19 Guidance: Reopening phase for public and community swimming pools | June 2020 | <https://coronavirus.delaware.gov/wp-content/uploads/sites/177/2020/06/Public-and-Community-Swimming-Pools-Phase-2.pdf> | U.S. | English | Pool | Yes |
| 8 | Public Health Agency of Canada (PHAC) |  |  |  | Canada | English | Beach and pool (outdoor activities, including [...] outdoor pools and beaches) | No |
|  |  | 1- Risk mitigation tool for outdoor recreation spaces and activities operating during the COVID-19 pandemic | September 2020 | <https://www.canada.ca/en/public-health/services/diseases/2019-novel-coronavirus-infection/guidance-documents/risk-mitigation-tool-outdoor-recreation-spaces-activities-operating-covid-19.html> |  |  |  |  |
|  |  | 2- Coronavirus disease (COVID-19): Measures to reduce COVID-19 in your community | March 2021 | <https://www.canada.ca/en/public-health/services/diseases/2019-novel-coronavirus-infection/prevention-risks/measures-reduce-community.html> |  |  |  |  |
| 9 | Food and Environmental Hygiene Department | COVID-19 -recommended measures to be adopted for swimming pools | July 2020 | <https://www.fehd.gov.hk/english/licensing/guide_general_reference/Prevention_and_Control_of_Disease_Regulations_SPL_20200706.html> | China | English | Pool (pools and spa) | Yes |
| 10 | Maryland Department of Health (MDH) | 1- Directive and order regarding swimming pools | May 2020 | <https://phpa.health.maryland.gov/Documents/20.20.06.10.03%20-%20MDH%20Order%20-%20Amended%20Pools.pdf> | U.S. | English | Pool | Yes |
|  |  | 2- Interim Guidance for Maryland Public Pools and Spas | May 2020 | <https://health.maryland.gov/qahealth/SiteAssets/documents/Interim%20Guidance%20for%20Maryland%20Public%20Pools%20and%20Spas%2021May2020.pdf> |  |  |  |  |
| 11 | New York State Department of Health | 1- Interim guidance for beach activities during the COVID-19 public health emergency | June 2020 | <https://www.governor.ny.gov/sites/governor.ny.gov/files/atoms/files/BEACHES_MASTER_GUIDANCE_05.19.20_FINAL.pdf> | U.S. | English | Beach (lake and ocean beaches, and shorelines) | Yes |
|  |  | 2- Interim Guidance for Pools and Recreational Aquatic Spray Grounds During the COVID-19 Public Health Emergency | June 2020 | <https://coronavirus.health.ny.gov/system/files/documents/2020/06/doh_covid19_pooladvisory_061120_0.pdf> |  |  |  |  |
| 12 | Food and Housing Division of the County of San Diego, Department of Environmental Health | 1- Public swimming pool operations during COVID-19 guidance | June 2020 | <https://www.sandiegocounty.gov/content/dam/sdc/deh/fhd/pool/covid19poolguidance.pdf> | U.S. | English | Pool (pools and spa) | Yes |
|  |  | 2- Coronavirus (COVID-19) Resources and Reopening Guidance | April 2021 | <https://www.sandiegocounty.gov/content/sdc/deh/deh_covid/deh_covid_fhd.html#pool> |  |  |  |  |
| 13 | Norwegian Institute of Public Health (NIPH) | 1- Sport, training and swimming | March 2021 | <https://www.fhi.no/en/op/novel-coronavirus-facts-advice/advice-and-information-to-other-sectors-and-occupational-groups/sport-and-organised-leisure-activities/> | Norway | English | Pool | Yes |
|  |  | 2- Advice for swimming pools and bathing facilities | April 2021 | <https://www.fhi.no/en/op/novel-coronavirus-facts-advice/advice-and-information-to-other-sectors-and-occupational-groups/swimming-pools-and-bathing-facilities/> |  |  |  |  |
| 14 | South Carolina Department of Health and Environmental Control (SCDHEC) | Interim guidelines for re-opening public swimming pools | May 2020 | <https://scdhec.gov/sites/default/files/media/document/Interim%20Guidelines%20for%20Reopening%20Public%20Swimming%20Pools.pdf> | U.S. | English | Pool | Yes |
| 15 | Georgia Department of Public Health | COVID-19 guidance – public swimming pools | June 2020 | <https://dph.georgia.gov/document/document/envhealthpoolguidance/download> | U.S. | English | Pool (pools, spas, and recreational water parks) | Yes |
| 16 | Baltimore Country Department of Health | Interim guidance for Baltimore county public pools & spas | May 2020 | <http://resources.baltimorecountymd.gov/Documents/Health/covid19/interimguidancepublicpoolspa.pdf> | U.S. | English | Pool (pools and spa) | Yes |
| 17 | Ohio Department of Health | Responsible restart Ohio: Local and public pools and aquatic centers | July 2020 | <https://coronavirus.ohio.gov/static/responsible/Pools-Aquatic-Centers.pdf> | U.S. | English | Pool (pools and aquatic centers) | Yes |
| 18 | State of Alaska | Swimming Pools Attachment P | May 2020 | <https://covid19.alaska.gov/wp-content/uploads/2020/05/05072020-Phase-II-016-Attachment-P-Swimming-Pools.pdf> | U.S. | English | Pool (pools and swim facilities, including facilities hosting swim clubs or teams, whether stand-alone or pools that are associated with a business offering other services such as a gym or hotel) | Yes |
| 19 | Office of the Arizona Governor | Guidance for pools | Not specified | <https://azgovernor.gov/sites/default/files/guidance_for_pools.pdf> | U.S. | English | Pool | Yes |
| 20 | Colorado Department of Public Health & Environment | Personal recreation | April 2021 | <https://covid19.colorado.gov/safer-at-home/recreation> | U.S. | English | Pool (indoor pools, outdoor swimming pools (any pool open to the public, including but not limited to municipal pools, homeowner association pools, pools at fitness centers, hot tubs, and developed hot springs)) | No |
| 21 | Government of Idaho (Idaho Department of Health and Welfare-Idaho Rebounds) | 1- Reopening Guidance for Idaho After COVID-19 | Not specified | <https://healthandwelfare.idaho.gov/Health/EnvironmentalHealth/HealthyCommunities/Pools_COVID-19/tabid/4752/Default.aspx> | U.S. | English | Pool (public pools or other recreational water facilities, indoor pools, and outdoor pools (outdoor community pools, splash parks, and waterparks)) | Yes |
|  |  | 2- Protocols for Indoor Gyms and Recreational Facilities | Not specified | <https://rebound.idaho.gov/wp-content/uploads/Protocols_IndoorGymsB1026-01.pdf> |  |  |  |  |
|  |  | 3- Protocols for Outdoor Pools, Splash parks, and Waterparks | Not specified | <https://rebound.idaho.gov/wp-content/uploads/Protocols_OutdoorPoolsC1026-02.pdf> |  |  |  |  |
| 22 | Maine Department of Economic and Community Development | 1- COVID19 Prevention Checklist Industry Guidance -Phase 2: Day Camps and Summer Recreation Programs | May 2020 | <https://www.maine.gov/decd/sites/maine.gov.decd/files/inline-files/COVID%20Checklist%20for%20ME%20Phase%202%20Day%20Camps.pdf> | U.S. | English | Pool (pools and other aquatic activities (hot tubs, spas, water playgrounds, or water parks); ocean, lakes and ponds) | No |
|  |  | 2- Lodging - COVID19 Prevention Checklist Industry Guidance | March 2021 | <https://www.maine.gov/decd/checklists/lodging> |  |  |  |  |
| 23 | Minnesota Department of Health | Reopening of Public Swimming Pool and Aquatic Facilities | April 2021 | <https://www.health.state.mn.us/diseases/coronavirus/poolreopen.pdf> | U.S. | English | Pool (public swimming pools and spa) | Yes |
| 24 | Montana Municipal Interlocal Authority | Montana Municipal Interlocal Authority Risk Management Bulletin | May 2020 | <https://mmia.net/wp-content/uploads/Municipal-Pool-Reopening-After-COVID.pdf> | U.S. | English | Pool (municipal and pools in gyms) | Yes |
| 25 | State of Nevada Executive Department | Roadmap to Recovery for Nevada: Phase 2 | June 2020 | <https://gov.nv.gov/uploadedFiles/govnewnvgov/Content/News/Press/2020_attachments/RoadmapToRecovery-Phase2GuidanceNoLinksVF.pdf> | U.S. | English | Pool (“public aquatic venues”; apartment complexes; home owners associations (HOAs); membership clubs including gyms or other privately owned aquatic centers accessible to the public through paid memberships or fees; schools; and hotels, motels, resorts, time-shares, and other guest lodging facilities) | No |
| 26 | North Carolina Department of Health and Human Services | Interim Guidance for Public Pools and Spas | March 2021 | <https://covid19.ncdhhs.gov/media/37/open> | U.S. | English | Pool (public pools including municipal, school, hotel, motel, apartment, boarding house, athletic club, or other membership facility pools and spa) | Yes |
| 27 | North Dakota Response | ND smart restart guidelines for recreational pools and water playgrounds | May 2020 | <https://ndresponse.gov/sites/www/files/documents/covid-19/ND%20Smart%20Restart/Pool%20and%20Water%20Playground/SmartRestart%20Pool.pdf> | U.S. | English | Pool (recreational pools and water playgrounds) | Yes |
| 28 | Oklahoma State Department of Health | COVID-19 Public Bathing Reopening Guidance | April 2020 | <https://www.ok.gov/health2/documents/Public%20Bathing%20ReOpening%20Guidance.pdf> | U.S. | English | Pool (pools, hot tubs and spas) | Yes |
| 29 | Tennessee-Office of the Governor | Tennessee Pledge | October 2020 | <https://www.tn.gov/content/dam/tn/governorsoffice-documents/covid-19-assets/Pledge_General.pdf> | U.S. | English | Pool (any indoor or outdoor aquatic venue or facility, including community, members-only, housing complex, hotel, waterpark, and exercise facility swimming pools) | Yes |
| 30 | Texas State | 1- Checklist for parks/beaches/ bodies of water | March 2021 | <https://open.texas.gov/uploads/files/organization/opentexas/OpenTexas-Checklist-Parks-Beaches-Bodies-of-Water.pdf> | U.S. | English | Pool and beach (parks, beaches, rivers, and lakes) | Yes |
|  |  | 2-Checklist for waterpark operators | March 2021 | <https://open.texas.gov/uploads/files/organization/opentexas/OpenTexas-Checklist-Waterpark-Operators.pdf> |  |  |  |  |
| 31 | Washington State Department of Health | COVID-19 Guidance: Water Recreation Facilities | March 2021 | <https://www.doh.wa.gov/Portals/1/Documents/1600/coronavirus/ReopeningWaterRecreationFacilitiesCOVID19.pdf> | U.S. | English | Pool | Yes |
| 32 | West Virginia Strong (The Comeback) | A Guide for Safely Reopening Bowling  Alleys and Other Indoor Recreation Facilities | March 2021 | <https://governor.wv.gov/Documents/Covid%20Week%205/2020.05.22%20Guidance%20for%20Bowling%20Alleys,%20Pool%20Halls,%20Ice%20and%20Roller%20Rinks,%20and%20Similar%20Facilities%20of%20Indoor%20Amusement.pdf> | U.S. | English | Pool (and other indoor recreation facilities including Bowling Alleys and others) | Yes |
| 33 | Queensland Aquatic Industry Alliance | Swimming Pool and Aquatic Centre Industry COVID Safe Plan Stage 3 and Onwards | December 2020 | <https://www.covid19.qld.gov.au/__data/assets/pdf_file/0025/134935/covid-safe-industry-plan-swimming-pool-and-aquatic-centre.pdf?nocache-v5> | Australia | English | Pool | Yes |
| 34 | Government of Western Australia | COVID Safety Guidelines – Sport and Recreation Phase 2 Version 1.0 | June 2020 | <https://www.wa.gov.au/sites/default/files/2020-05/COVID%20Safety%20Guidelines%20Phase%203%20-%20Sport%20and%20Recreation.pdf> | Australia | English | Pool (sport and recreation (swimming pools and aquatic facilities)) | No |
| 35 | International Association for Sports and Leisure Facilities (Germany) | Recommendations of IAKS Germany for the phased re-opening of municipal swimming pools | April 020 | <https://iaks.sport/sites/default/files/downloads/Best%20practices/2020-04/IAKS%20Germany%20Recommendations%20for%20opening%20of%20public%20pools%20step%20by%20step.pdf> | Germany | English | Pool (municipal swimming pools) | Yes |
| 36 | Coatian Institute for Public Health | Croatia publishes recommendations for beachgoers | May 2020 | <https://www.croatiaweek.com/croatia-publishes-recommendations-for-beachgoers/> | Croatia | Croatian | Beach | Yes |
| 37 | Japan Sports Agency | [Guidelines for preventing the spread of infection for the resumption of social education facilities] | February 2021 | <https://www.mext.go.jp/sports/content/20200514-spt_sseisaku01-000007106_1.pdf> | Japan | Japanese | Pool | No |
| 38 | Sport New Zealand | Play, active recreation and sport at Alert Level 2 | May 2020 | <https://sportnz.org.nz/assets/Uploads/Play-Active-Recreation-and-Sport-at-Alert-Level-2.pdf> | New Zealand | English | Beach | No |
| 39 | European Waterpark Association (EWA) | Two-sate-plan of the European waterpark association E.V. for the reopening of waterparks and spas | Not specified | <https://iaks.sport/sites/default/files/downloads/Best%20practices/2020-04/EWA_Two-Stage-Plan%20of%20the%20European%20Waterpark%20Association%20for%20the%20Reopening%20of%20Waterparks%20and%20Spas.pdf> | Europe | English | Pool (waterparks /pools, spa) | Yes |
| 40 | VHF Switzerland | [Protection concept for indoor and outdoor pools of the VHF after reopening after the "Corona closing time"] | June 2020 | <https://www.vhf-gsk.ch/data/index.php/component/jdownloads/send/13-vhf-news/57-vhf-schutzkonzept-bei-wiedereroeffnung-nach-coroan> | Switzerland | Swiss | Pool (indoor and outdoor pools) |  |
| 41 | USA Swimming | Facility re-opening messaging and planning | Not specified | <https://cdn.swimswam.com/wp-content/uploads/2020/05/facility-reopening-plan-guidelines.pdf> | U.S. | English | Pool | Yes |
| 42 | Pool & Hot Tub Alliance | 1-Practice tips for maintaining and opening public pools and hot tubs | Not specified | <https://www.cabq.gov/environmentalhealth/documents/opening-pools-and-hot-tubs.pdf> | U.S. | English | Pool (pools and hot tubs) | Yes |
|  |  | 2- Coronavirus toolkit for pool and hot tub professionals |  | <https://www.phtacoronaupdate.com/PHTACORONAUPDATE/assets/File/4891%20PHTA%20COVID%20Complete%20Guide%20444-V12.pdf> |  |  |  |  |
|  |  | 3- The health and safety benefits of properly maintaining your pool and hot tub |  | <https://www.neha.org/sites/default/files/eh-topics/water-quality/PHTA-Operator-COVID-Flier.pdf> |  |  |  |  |
| 43 | Finnish Association for Swimming Education and Lifesaving (SUH) | SUH Guidelines for Swimming Pool Operators for the Prevention of Coronavirus Infections 1- [Swimming pool safety during the corona epidemic - SUH view of the considerations to be taken into account when opening swimming pools] 2- [Beach safety during the corona epidemic - instructions for beaches administrators] | May 2020 | 1- <https://www.suh.fi/files/2824/Uimarantojen_turvallisuus_koronaepidemian_aikana_-_ohjeet_rantojen_yllapitajille_paivitetty_19.5.2020.pdf>  2- <https://www.suh.fi/files/2824/Uimarantojen_turvallisuus_koronaepidemian_aikana_-_ohjeet_rantojen_yllapitajille_paivitetty_19.5.2020.pdf> | Finland | Finnish | Beach and pool | Yes |
| 44 | Federal Ministry for Social Affairs, Health, Care and Consumer Protection of Austria | Recommendations for reopening facilities after Pool Hygiene Act (BHygG) and the Pool Hygiene Ordinance 2012 (BHygV 2012) | July 2020 | <https://www.sozialministerium.at/dam/jcr:a5229476-70f6-4578-8412-2afc763ce90d/20200519_Empfehlungen%20zur%20Wieder%C3%B6ffnung%20von%20Einrichtungen%20nach%20dem%20B%C3%A4derhygienegesetz%20(BHygG)%20und%20der%20B%C3%A4derhygieneverordnung%202012%20(BHygV%202012).pdf> | Austria | German | Pool (pools, baths, and saunas: artificial outdoor pools, baths on surface water, small bathing ponds, bathing water, indoor swimming pools, whirlpools, sauna facilities, warm air and steam baths | Yes |
| 45 | Associazione Nazionale Impianti Fitness e Sport | Protocollo delle misure per il contrasto ed il contenimento della diffusione del virus covid-19 nei centri sportivi condiviso dal governo/parti sociali/reoioni il 17 maggio 2020 [Protocol of measures to combat and contain the spread of the covid-19 virus in sports centers shared by government/social partners/regions] | May 2020 | <https://www.google.com/url?sa=t&rct=j&q=&esrc=s&source=web&cd=&ved=2ahUKEwjN1e7K9pfrAhWuHjQIHS_LA9QQFjAAegQIBBAB&url=https%3A%2F%2Fwww.europeactive.eu%2Ffile%2F832%2Fdownload%3Ftoken%3DbgxLpor9&usg=AOvVaw19_xKl7gmXXGT8OBfgn4_0> | Italy | Italian | Pool | No |
| 46 | Société Française d’Hygiène Hospitalière (SF2H) | Avis relatif au risque de transmission hydrique du SARS-CoV-2 dans l’eau des piscines publiques et leur environnement [Notice relating to the risk of water transmission of SARS-CoV-2 in the water of public swimming pools and their environment] | March 2020 | <https://www.sf2h.net/wp-content/uploads/2020/03/Avis-SARS-CoV-2-et-eau-de-piscine-SF2H-09.03.2020.pdf> | France | French | Pool (public swimming pools) | Yes |
| 47 | GSMS-SItI, Working Group on Movement Sciences for Health of the Italian Society of Hygiene Preventive Medicine and Public Health | Swimming Pool Safety and Prevention at the Time of Covid-19: A Consensus Document From GSMS-SItI | 2020 | - DOI: 10.7416/ai.2020.2368 | Italy | English | Pool (pools (consider a reopening for sport and physical activities)) | Yes |
| 48 | Arkansas Department of Health | Directive for Recreational Pool Reopening Proposal | April 2021 | <https://www.healthy.arkansas.gov/images/uploads/pdf/guidance_pools.pdf> | U.S. | English | Pool and beach (all types of recreational pools, including seasonal outdoor pools for state parks, hotels, motels, apartments, subdivision/POAs., indoor pools, municipal and community pools, splash pads, aquatic centers, commercial water parks, as well as swim beaches) | Yes |
| 49 | Connecticut State | 1- Governor Ned Lamont: Best Practices for Reopening | March 2021 | <https://portal.ct.gov/-/media/DECD/Covid_Business_Recovery-Phase-2-1/CTReopens21_Sports_FitnessCenters319.pdf> | U.S. | English | Pool and beach (sports pools, public pools, beaches and swimming areas) | No |
|  |  | 2- COVID-19 Guidance for Reopening Connecticut’s Recreational Areas and Activities | May 2020 | <https://portal.ct.gov/-/media/Departments-and-Agencies/DPH/dph/environmental_health/COVID-19/COVID-19_Reopening-Recreational-Areas-and-Public-Pools_051920_FINAL.pdf?la=en> |  |  |  |  |
|  |  | 3- Beaches and Swimming Areas – COVID 19 Guidance | May 2020 | <https://portal.ct.gov/-/media/Departments-and-Agencies/DPH/dph/environmental_health/COVID-19/COVID19_2020-Circular-Letter_Beaches-Start-Up_051920_FINAL.pdf> |  |  |  |  |
|  |  | 4- Start-Up of Outdoor Public Pools – COVID 19 Guidance | May 2020 | <https://portal.ct.gov/-/media/Departments-and-Agencies/DPH/dph/environmental_health/COVID-19/COVID19_Public-Pools_Start-Up_051920_FINAL.pdf> |  |  |  |  |
|  |  | 5- Guidance for Reopening Public Pools and COVID-19 | June 2020 | <https://portal.ct.gov/-/media/Departments-and-Agencies/DPH/dph/environmental_health/COVID-19/COVID19_Circular-Letter_Reopening-Public-Pools_061020_Signed.pdf> |  |  |  |  |
|  |  | 6- Reopening Guidance for Public Pools | June 2020 | <https://portal.ct.gov/-/media/Departments-and-Agencies/DPH/dph/environmental_health/COVID-19/COVID19_Reopening_Guidance_for_Public_Pools.pdf> |  |  |  |  |
| 50 | Ramya et al., 2021 | Resumption to Swimming Post COVID-19 Lockdown | 2021 | DOI: 10.7860/JCDR/2021/46392.14448 | India | English | Swimming pool (for athletes) | Yes |

Abbreviations: U.S.: United States

# **Appendix 4:** Detailed description of recommendations organized by topics and sub-topics (N=50)

*Note: Numbers in the tables below refer to the organization numbers displayed under the column titled “ID” in Appendix 3*

## **Ensuring social distancing (n=50)**

| **What to do** | **How to do it** |
| --- | --- |
| **Capacity control (n=44)** | |
| Limit capacity/number of people (n=38) to:   - Percentage capacity: 50% [5, 11, 18, 23, 25, 28, 31, 49], 60% [7], 75% [24, 26], 80% [9], or depends on county’s COVID-19 risk level ranging between 10 people to 100% [20] or 0% to 50% [13] - Persons per square meter (sqm) of surface area: 1 person per 2 sqm of pool surface area [33, 34], 1 person per 6 sqm of pool surface area [42, 44], 1 person per 25 sqm in small bathing ponds [44], 1 person per 10 sqm of water surface and surrounding areas or lawn areas [40], 15 persons per 100 m2 of net area in sea and freshwater [36], 3 persons per 2 sqm of outdoor water and 1 person per 1 sqm of indoor water [46], more than 2 m^2^ per person [50] - A fixed maximum number of persons: 50 persons [31] 100 persons (also subject to 1 person per 2sqm) [34], 200 persons (or half the number of lockers) in phase 1 – ‘during widespread social distancing’, increased to 400 persons (or 2/3 the number of lockers) in phase 2 – ‘during easing of regulations’ [35], 1 person in whirlpool bath [13] - A maximum number of persons according to: area and capacity in changing rooms, toilets and showers [13], determination by the staff [39], half the number of lockers in phase 1 (‘during widespread social distancing’), increased to 2/3 the number of lockers in phase 2 (‘during easing of regulations’) [35], size of the swimming pool and a risk assessment made by the administrator of the swimming pool [43], limit set by Governor or local jurisdiction [8, 10, 16, 22, 27, 31], authority having jurisdiction (AHJ) order and social distancing requirements, the facilities configurations, and square footage, zones, and lanes [42] - Capacity not specified [2,6, 8, 12, 14, 15, 17, 19, 21, 29] | Ways to limit number of guests at a given time (n=30):   - Reservation system [1, 6, 11, 12, 14, 17, 20, 22, 23, 26, 31, 33, 39, 42, 43, 45, 47, 49] - Sign-up board [12], sign-in/out sheet [16] - Pre-existing electronic capacity monitoring systems [20] - Front desk tracking for occupancy [24] - Phone app technology to alert patrons when the pool area is available based on schedule or occupancy [26] - Limiting number of admissions per day [29] - Selling in advance tickets via the internet/advance booking offices [33, 44], so that only few tickets have to be bought on site [44] - Establishing time limits [5, 17, 23, 27, 40], limiting length of activities [49], encouraging guests minimize time spent in the facility by arriving and leaving wearing their swimsuit [25, 49], discouraging on-deck time for pool users before/after they have completed their intended activity [33], creating a low-cost short-stay ticket (1, 1.5 or 2 hours), and raising other ticket prices significantly [35] - Limiting group sizes to 10 persons [6, 11, 17, 27, 30, 40], 20 persons for outdoor sporting-based activities [33], 30 persons^^[[3]](#footnote-3)^^ [40] - Prohibiting or reducing the number of spectators for swim lessons, competitions, events [18, 21, 29, 33] - Monitoring capacity limits and access through screener at pool entrance and video for non-guarded pools [23, 42] |
| Limit number of users of specific facilities (n=19):   - Restrooms/sinks [1, 8, 11, 17, 22, 26, 32, 37, 40, 42] - Showers [8, 17, 32, 39, 40, 45, 49] - Changing areas [11, 37, 39, 43, 45] - Locker rooms [1, 11, 20, 21, 42], lockers [35, 39, 43] - Communal spaces [14] - Play features [27] - Rest spaces [37] - Cloakroom boxes [40] | Ways to limit number of users of specific facilities at a given time (n=18):   - Reducing number available [39, 40, 43], opening in alternate manner (i.e. ’every other’) [13, 17, 26, 35, 39, 40, 45, 49], rearranging [23], closing a series [20], reducing number/capacity to half [8, 11], making every third available [21, 40], closing every third [17, 39, 49] - Flagging when occupied [11] - Staggering entry/use [1, 14, 15, 23] - Operating at intervals [27] - Limiting number of issued keys [39] - Closing stalls [8] |
| **Distancing measures (n=49)** | |
| Ensure social distancing (n=49) of:   - 6 feet [1, 3, 5, 6, 7, 10, 11, 12, 14, 15, 16, 17, 18, 19, 20, 21, 22, 23, 24, 25, 26, 27, 28, 29, 30, 31 32, 42, 48, 49, 50] - 1 meter [13, 45], 1.5 meters [9, 33, 34, 36, 39], 2 meters [8, 37, 40, 43] - 10 feet while swimming laps [18], 1 meter in changing rooms [13, 18, 44], showers [18, 44], attractions and diving platforms with waiting times [44], whirlpools (at most every second place is filled) [44], between swimmers and trainers [13], 6 feet between lanes [50] - Greatest extent possible [29] - More than two meters at seashore^^[[4]](#footnote-4)^^ [2] - Distance between individuals not specified [4, 31, 35, 38, 41, , 47]   Distancing may not necessarily be observed by parts of the same household/guests staying in a shared apartment [1, 5, 7, 10, 11, 12, 15, 20, 21, 23, 25, 30, 31, 44, 48, 49]  In addition to patrons/swimmers, distance should be observed by employees [6, 11, 15, 16, 17, 18, 19, 20, 21, 22, 26, 29, 30, 31, 32, 33, 35, 36, 42, 43], lifeguards [5, 11, 19, 26, 49] and spectators [8, 29, 37, 42, 49, 50] | Use of physical barriers (n=24), such as:   - Chairs and tables on the deck [1, 17, 20, 32, 49, 50] - Separation barriers (e.g. plexiglass separator, safety glass or foil, partitions, plastic shielding wall, strip curtains, ‘sneeze guards’, other impermeable dividers) at cashier [4, 31, 37, 39, 40, 42, 43], at reception [13], in ticket booths [11, 29], information, and beach booths and service windows [11], for gate attendants [26], between workstations [11], in the entrance area [35], at front desk [31, 49] - Plastic partitions [5], partitions in changing rooms [11] - Barriers and protective shields [17] - Orange cones [5] - Rubber mats [5] - Barricade tape (similar to that used at airport check-in) [39] - Other easily cleanable products [5] - Ropes [11], buoys and floating pool ropes to mark off lanes or areas of pool [24], flotation devices [23] - ‘Physical barriers’ [8, 11, 17, 23, 29], ‘physical cues’ [11, 15, 27, 50]   Physical barriers must not present a health hazard (e.g. impede air flow, obstruct supervision) [11, 31] |
|  | Use of visual cues (n=30), such as:   - Floor or wall markings [8, 11, 13, 15, 23, 26, 29, 31, 33, 35, 39, 40, 41, 42, 44, 48], such as sports lime or chalk [8] - Tape [1, 5, 6, 17, 20, 25, 32, 49] - Lane lines in water [1, 17, 20, 29, 32, 49, 50] - Signs [5, 33,34, 50], directional arrows [24, 29, 34] - Stickers [14, 43, 50] - Trail markers [8] - Flags [11] - ‘Visual guidelines’ [5], ‘visual cues’ [15, 17, 20, 27, 50] |
|  | Change layout (n=38) of:   - Deck [1, 3, 5, 10, 14, 15, 16, 17, 23, 24, 25, 26, 27, 29, 32, 36, 40, 45, 49] - Furniture (e.g. chairs/seats, blankets, recliners/loungers, tables) [5, 8, 9, 11, 12, 13, 17, 19, 21, 22, 23, 24, 25, 26, 28, 29, 31, 33, 34, 35, 36, 39, 40, 42, 43, 45], 10 feet between beach blankets and chairs [11], 2 meters between recliners and seats [35] - Distribution of positions to be assigned to bathers [4] - Work stations or areas (e.g. lifeguard chairs and towers) and employee seating areas [11, 50] - Waiting areas (e.g. reduce number of seats [19, 33, 39, 42, 45], limit to people with disabilities to avoid the stay [44]), service areas, and break rooms [19] - Seats during sports events (i.e. widely spaced) [50] |
|  | Manage traffic flow of individuals (n=20)   - Separate entry and exit points to the facility [1, 11, 18, 23, 26, 27, 33, 42, 45, 49] - Implement one-way traffic/unidirectional flow of person traffic [1, 5, 8, 11, 17, 18, 23, 27, 33,34, 44, 47, 49] - Separate entry and exit points to restrooms and showers [26, 40] - Direct flow of patrons and employees through facility to reduce contact, where possible, including spacing or staging lines for slides, rides and other attractions [21] - Create social distancing plan [27, 32], “guest flow” plan [29] |

| **Ban/restrict access to specific areas (n=24)** |
| --- |
| - Locker rooms [15, 41], changing rooms [12, 13, 18, , 39, 41], shower areas without partitions [40], - hot tubs [5, 10,18, 25, 29, 39], steam pools [31, 39], lazy rivers [10] - All indoor slides or slides not directly exposed to the sun [21], water playgrounds, or water parks [22], water games [45], playgrounds and high-contact water play areas (e.g., splashpads) [29], water play features that cannot be safely used with adhering to the six-foot minimum distancing [27], kiddie pool or other congregate areas of pools (consider prohibiting or strict monitoring) [21] - Seating areas, tables, chair (if safe distances are not achievable, or regular sanitizing of these areas is not possible) [17] - Spectator stand [9, 49], grandstands [45]; allowed if limited to one parent/guardian per swimmer along with face mask [49] given it can be thoroughly cleaned and disinfected before and after every use, and distance can be maintained [49] - Waiting areas [28] - During phase 1 (‘widespread social distancing’), restaurants and outdoor swimming pools will remain closed (indoor swimming pools opened for school games, organized sport and for individuals); during phase 2 (‘easing of regulations’), outdoor swimming pools are re-opened with limited capacity [35] - Concession businesses [11] - Small dryland rooms [41] - Check-in counters (unless touchless) [49] - Non-essential features/indoor spaces or common areas that encourage people to gather [8, 25] - ‘Certain areas/equipment’ [32], ‘common areas’ [49], “communal areas” [31] |
| **Ban/restrict access to specific activities (n=24)** |
| - swimming lessons that require the instructor to have physical contact with the patron [21, 35, 49], swimming lessons during phase 1 (‘widespread social distancing’) [35] - Pool activity limited to lap swimming only [20, 21], to single person per swimming lanes [22, 26, 49] - pool activities such as water volleyball or basketball [27, 29], water polo [29, 31] - Events [15, 16, 35, 45], gatherings [16], classes [15], group events, gatherings, meetings or classes if social distancing cannot be maintained [14, 19, 21, 22], with any gathering more than 10 allowed [22] - Consider how some activities could be conducted outside and limit use of indoor facilities [21] - Consider impact on programs – recreational swim, water exercise, lap swim, swim lessons, swim team practices; develop a plan on if and how for each [42] - Features that make physical distancing difficult (e.g., lazy river propulsion, wave machines in wave pools, and other play features that take away control of movements from patrons) must be turned off, removed, or adjusted (e.g., low flow setting) so that patrons can maintain control of their body movements [31]   *Activities allowed under certain conditions:*   - Open swim allowed if in in-pool interactions are reduced [20] - Aquatic classes allowed if distancing can be maintained [7, 39, 43], if shared equipment is disinfected after use [8], if do not require the instructor to have physical contact with the patron and if distancing can be maintained [21] - Spray grounds and water features allowed if distancing can be maintained [12] - Swimming classes allowed if   - Number of participants is limited [14, 23, 29], is limited to 10 persons [24], 25 students [23]   - Distancing can be maintained [21, 35, 43]   - Instructors teach from pool deck [12]   - Do not require the instructor to have physical contact with the patron (e.g. swim team sessions with older children) [21, 49]   - Unnecessary close contacts are avoided [43]   - Hot tubs allowed if maximum of one user at a time [13, 49], if not possible to achieve a minimum of 1 meter distance between users [13], if distances are marked between the lying areas [39] while using face masks [22] - Events or classes allowed if number of participants if limited, social distancing is ensured, sharing of equipment or of items that are difficult to clean is discouraged, nonessential visitors, volunteers, and activities involving external groups or organizations are limited, traveling for events (i.e. swim meets) is limited [19] - Parties allowed with time limits [14] - Life-saving courses allowed if distancing can be maintained [13] - Torrent pools, toboggans and soft slides allowed if distancing and recommended water parameters can be maintained [45] - Competitive sports allowed if physical distancing is respected, capacity is considered, and teams are not mixed. All attendees, staff and any contractors contact details must be recorded at the outset of every competition and be retained for at least 56 days [33] |
| **Modify schedule to reduce individuals’ interaction (n=19)** |
| - Stagger access times for different groups [8, 11, 20, 23, 25, 26, 31, 43]; e.g. create A/B teams [11] - Establish a schedule with time slots for various activities [26, 32, 42], and stagger the start times of possible group activities [1, 43, 49] - Adjusting beach hours/adopt strategic hours of operation or flexible closure criteria [11] - Consider offering special operating hours for at risk populations [7]   Employees:   - Stagger shift start/stop times and break times to minimize contact across employees [1, 15, 17, 22, 26, 34, 49] - Divide staff by duty roster into groups that have no contact with each other [1, 17, 35] |
| **Pool and area monitoring (n=18)** |
| - Intensify the pool and water supervision [39] - Deploy designated onsite manager or other responsible person [7], monitor on deck [7], staff member [15, 24, 27, 30, 33, 35, 36] for monitoring purposes - Deploy additional monitoring staff [9, 35, 49] - Monitoring   - Capacity limits [6, 23, 27, 33, 35, 40, 42]   - Social distancing [6, 11, 15, 24, 27, 35, 36, 48]   - Vehicle occupant counts [11]   - Beach access points [11] - Train staff in COVID-safe requirements to enable them to monitor patronage numbers and assess capacity limits based on persons per sqm [33] - Outdoor pool area must be fenced or closed to allow monitoring number of guests at entry and exit [8]; restrict pool access to fewer entrances as needed to control access and overcrowding [48] - In the event of non-compliance, staff will respond earlier than usual with expulsion from the facility [35] |
| **Swimming-specific distancing measures (n=14)** |
| - Limit pool activity to lap swimming only [20, 21]; if open swim is permitted, make efforts to reduce interactions between people [20] - Limit swimming to single person per lane [22, 26, 49], especially high-risk patrons [31] - Two swimmers may use the same lane only if they do not enter, exit or rest within 6 feet of each other [8, 23, 26, 31], or are from the same household [8]; 10-foot social distancing should be maintained while swimming laps [18] - Consider lane reservations for families [14] - Discourage lap swimmers from gathering/resting at the end of lanes [1, 33, 38] - Stagger lap swims, practices and lessons at opposite ends or corners of the pool [42] - Limit to a maximum of 4 swimmers is permitted for supervised competitive swimming team practices [31, 49], given they stay the same four swimmers throughout the swimming season and do not congregate at the end of the pool [49] |
| **Other measures (n=17)** |
| - Parental supervision for children/minors [12, 14, 29, 39, 45, 50] - Ensure that changing layout still provides a clear deck space of 4 feet around the pool perimeter, as required by law for emergency rescue [12, 15] - Ensure that capacity allows for proper social distancing on the pool deck in the event of a fecal incident or other life-safety situation where bathers may need to exit pool and remain on deck or evacuate to another location [23] - Train staff about social distancing [4, 5, 6, 18 22] and monitoring and assessing capacity [33] - Staff training should be conducted virtually [4, 5, 10, 15, 16, 22, 33], or social distancing should be ensured during training [4, 5, 10, 15, 16] - Limit staff and patrons to people who live in the local geographic area (e.g., community, city, town, or county) to reduce risk of spread from areas with higher levels of COVID-19 [1,15] |

## **Ensuring personal hygiene (n=45)**

| **Promoting personal hygiene practices (n=35)** |
| --- |
| - Encourage patrons and staff to frequently wash their hands (n=33)   - With soap and water [1, 5, 8, 14, 17, 18, 19, 21, 26, 27, 28, 29, 31, 32, 33, 37, 42, 45, 46, 49, 50]   - For a minimum of 20 seconds [1, 5, 6, 8, 14, 17, 19, 21, 26, 28, 31, 33, 49] or 30 seconds [37]   - Before and after entering the pool [2, 13]   - No further details [4, 7, 8, 9, 15, 16, 22, 23, 24, 30, 38, 43] - Protocol for the staff while entering and exiting the workplace: use hand sanitizer or wash their hands upon arrival and departure and often throughout the day [21, 26, 30] and between interactions with visitors [30] - Encourage patrons/employees to avoid touching their face (eyes, nose, and mouth) with unwashed hands [6, 7, 8, 9, 13, 17, 21, 29, 31, 43, 50] - Encourage respiratory etiquette, such as covering the mouth while coughing, and sneezing in the elbow [1, 7, 8, 13, 14, 15, 16, 17, 21, 26, 28, 29, 30, 31, 37, 38, 50] - Patrons should shower prior to arriving [1, 49], prior to entering the pool [50] - Train staff on new hygiene procedures [10, 22, 30] - Children may need supervision to ensure effective hand hygiene is performed [1, 8] and to prevent ingestion or sanitizer splashing or getting into their eyes [1] |
| **Facilitating personal hygiene practices (n=27)** |
| - Access to hand sanitizer dispensers/washing stations [4, 6, 8, 9, 10, 11, 17, 18, 19, 21, 23, 25, 26, 29, 30, 31, 32, 33, 36, 37, 38, 39, 40, 42, 49, 50] at the entrance [9, 17, 21, 23, 26, 34, 39, 40, 42, 49], cash registers [39], “key locations” [23], “easily accessible locations” [4], visible location [8, 36], in toilet areas [33], locker rooms [21] “around the pool” [19], “in staff areas” [29], “upon entry to the food area” [30], at common areas [49], near exits [42], at waiting areas [42], locations where people have direct interactions [17] and near highly touched surfaces [17, 49] - Check and frequently refill the dispensers of sanitizers [10, 17, 21, 26, 39] |
| **Ensuring adequate hygiene supplies (n=33)** |
| Supplies that should be available are:   - Soap [1, 5, 10, 11, 15, 16, 18, 19, 21, 26, 27, 29, 30, 31, 32, 33, 34, 37, 38, 42, 49] - Hand sanitizer [1, 6, 7, 8, 9, 10, 15,16, 17, 18, 19, 20, 25, 27, 29, 30, 31, 32, 33, 34, 37, 38, 49] - Alcohol-based hand rubs [1, 5, 6, 8, 11, 21, 23, 29, 33, 36, 50] with 60% alcohol [1, 5, 6, 8, 11, 14, 15, 23, 29, 31, 33, 38, 50], 45% alcohol [36], 70% iso-propanol [33] - Disinfectant [18, 26, 27], disinfecting wipes [5, 26, 30, 49] - Paper towels/tissues [50] instead of regular towels [1, 5, 11, 16, 18, 26, 33, 37, 42, 43, 49] |
| **Ensuring safe payment procedures (n=19)** |
| - Implement a system in which relief workers rotate into the cashier station to allow cashiers to leave the station to wash their hands regularly [27] - Ensure staff sanitize hands after each customer contact (actual physical contact, e.g. money exchange) [30, 33] - Implement touchless/contactless payment methods [11, 12, 15, 21, 27, 29, 30, 31, 33, 37, 39], cashless payments [18, 29, 32, 34, 35, 40, 43] and receipt-less transactions [18], contact process such as online reservations, timed-ticketing, permit/sticker issuance [5], if not possible, have hand hygiene supplies available for use after each transaction [11], and ensure that hands are washed with soap and water, or a hand sanitizer is used [34] - Clean and disinfect payment devices on an ongoing basis [49] |

## **Using personal protective equipment (PPE) (n=38)**

| - Wearing of face covering (mask or cloth face-covering) (n=32); by:   - Patrons [1, 3, 5, 6, 7, 8, 9, 10, 11, 13, 14, 16, 17, 18, 19, 20, 21, 22, 23, 26, 29, 30, 31, 32, 37, 49, 50]   - Staff [3, 4, 5, 6, 10, 11, 15, 16, 17, 18, 19, 20, 21, 22, 23, 25, 26, 27, 28, 29, 32, 42, 49, 50], reception staff [37, 40], cash register staff [40], gate attendants [26], those at a sign-in desk or snack bar [32]   - Lifeguards [3, 4, 5, 11, 16, 42, 49]   - Instructors of swimming classes [23, 25], coaches [49]   - Spectators [49] - Wear face covering (n=17)   - When physical distancing cannot be achieved [1, 6, 7, 8, 9, 11, 13, 15, 22, 28, 29, 30, 33, 38, 48, 50]   - “To the maximum extent practicable” [3, 25] - Do not wear face covering (n=20)   - In the water [1, 3, 5, 6, 7, 9, 10, 11, 14, 15, 16, 17, 18, 19, 22, 23, 26, 30, 31, 42, 49, 50]; as wet masks can cause difficulty breathing [3, 15, 17, 19, 23, 48, 49, 50] and can induce panic, especially in children [3] and wet masks do not slow the spread of COVID-19 as well as dry masks [1]   - On children under two years old [1, 3, 7, 8, 14, 32] or under the age of 5 [21]   - On people with difficulty breathing [1, 8, 14, 32]   - On people who cannot remove the mask themselves [1, 8, 14, 32]   - If contrary to individual’s health or safety due to medical conditions [21, 49]   - In high temperature and humidity [37] - Use of gloves by staff when cleaning [11, 33, 49], when handling towels [17, 19, 32, 50], where possible [21], by reception staff [40]; use of eye protection when using cleaning chemicals [49] or in case of splashing [33] - PPE should be put on before entering and cleaning areas that have been used by a person with suspected or confirmed COVID-19 infection; including disposable gloves, disposable apron or other protective garment, protective eyewear [33] - Wear of PPE by staff and patrons [32, 47]; create a plan for PPE [32]; wear of PPE is in line with Safe Work Australia and Guidelines for Safe Pool Operations (GSPO) recommendations [33] - Train on the use of PPE [1, 17, 22, 31,32, 42] - Swim instructors, lifeguards and attendants, should wear a face shield with cloth attachment that is made of waterproof or quick drying material (e.g., Tyvek, Badger Shield, Humanity Shield) [31] - Provide containers such as Ziploc bags on site or recommend patrons to bring their own containers to store their face coverings while not being used [31] - Make face coverings available on site for patrons who forget to bring their own, who lose theirs, or whose masks get damaged [31] - Encourage everyone to bring a second (or extra) cloth mask in case the first one gets wet [1] |
| --- |

## **Eating and drinking (n=25)**

| **Eating (n=23)** |
| --- |
| Food services (n=10)   - Close snack bars unless food service guidance can be followed [26] - Snack bars are permitted but should follow the guidelines for Restaurant Dine-in Operations [48] - Must be limited to take-out ‘in Phase 1’ [5] - Must be limited to prepackaged foods only [22, 25, 39, 49]; individually packed for each employee and/or contractors [30]; sales of only prepackaged food and drink does not have to follow Sector Rules for Restaurants [49] - “All open food and drinks must be served by staff” [39] - If food service will be provided, determine products permitted, where food may be eaten, and preparation and packaging of products [42] - Allowed to open, but must follow the Restaurant/Bar/Food Truck Smart Restart Protocols [27], follow the same recommendations as for hospitality facilities [36], follow Sector Rules for Restaurants [17, 49] - Use disposable menus (new for each patron), or clean and disinfect reusable menus after each use [30] - Do not leave condiments, silverware, flatware, glassware, or other traditional table-top items on an unoccupied table [30] |
| Sharing food (n=7)   - Discourage patrons from sharing food with persons that are not a part of their household [15, 16, 50] - Prohibit shared food and beverages among employees (e.g. buffet style meals) [11, 22] - Encourage employees to bring lunch from home [8] - Discontinue operations that require customers to use common utensils or dispensers (e.g. lids and sugars for hot beverages) [33] |
| Social distancing (n=11)   - Close benches, picnic tables, and designated congregate seating areas [11]; close restaurants in phase 1 (‘during widespread social distancing’) [35] - Distancing of tables   - Design seating areas to ensure 6 feet distancing [15], 1.5 meters between the individual tables [33, 39]   - Space benches and picnic tables [8]   - Reduce the number of tables/chairs provided in food and beverage service areas and adjust the configuration [33]   - Modify seating arrangements to meet the maximum group size requirement and social distancing requirement [33] - Distancing of individuals   - Maximum of 2 people permitted at the tables (except for families) [39]   - More than four people can sit at one table; however, a person must provide his/her contact details to the operator [40]   - Social distancing of patrons in all food service areas [42] and while drinking and dining-in [33]   - Groups maintain at least 6 feet of distance from other groups at all times, including while waiting to be seated in the food area [30]; set up different areas for food ordering and collection, and where practical, separate entry and exit paths [33]   - Restrict occupancy at restaurant or dining areas to be in accordance with applicable guidelines for restaurants [32]   - In phase 2 (‘easing of regulations’), the seating in the restaurants is to be arranged in such a way that people sit together in twos and that household groups such as families, can sit at a few larger tables, with appropriate marking [35]   - Discontinue use of shared tables among non-family units [26]; household groups may sit closer together than 6 feet at tables [48]   - Reserve adequate space for employees to observe social distancing while eating meals [8]   - Distance markings of 2 meters should be made in front of the vending machines [40] |
| **Drinking (n=12)** |
| - Pool bars are still allowed to serve drinks to-go but cannot designate seating or standing areas as lounge areas [39] - Only packaged drinks to be taken [39] - Encourage visitors to bring their own water/fluids [8, 12, 33, 49] - Clean and disinfect water bottle refill stations on an ongoing basis [49] and self-service dispensers for coffee, drinks, juice [22] - Drinking fountains [1, 7, 9, 11, 12, 16, 23, , 39, 49]   - Suspend use of drinking fountains [9, 12, 22, 39]; water fountain use restricted to water bottle filling stations only [49]   - Keep the drinking fountains functional [1, 7, 11, 16, 23], if a touchless activation system [11, 22] or foot pedal is installed [11]   - Disinfect drinking fountains every 15 minutes to 2 hours with an EPA-approved disinfectant [8] |

## **Maintaining the pool (n=33)**

| **Disinfectants (n=22)** |
| --- |
| Recommended disinfectant agents (n=20)   - Chlorine [1, 2, 3, 5, 9, 10, 12, 16, 18, 19, 21, 22, 23, 28, 34, 39, 41, 45, 46, 49] - Bromine [1, 3, 5, 18, 21, 22, 23, 28, 49] - Disinfectants listed by Environmental Protection Agency (EPA) [5, 34] - Disinfectants suggested by the venue designer [5] - Environment-friendly disinfectant for disinfection of natural floors (i.e. sand of beaches), although not recommended [2] |
| Recommended disinfectant concentration level (n=9)   - Free chlorine: 1 ppm [49], at least 1 ppm (ideally 2-4ppm) [40], 1-3 ppm [2, 3], 1-5 ppm [, 28], 2-10 ppm [12], 7-7.5 mg/L [45] or an average of 2 ppm [41] - Bromine: 3 ppm [49]2-4 ppm [28], - Set the thresholds at the maximum values allowed and do not only achieve the minimum requirements [47] |
| **Maintenance parameters (n=6)** |
| - pH: 6.5-7.5 [45], 7.1-7.6 [2], 7.2-7.8 [3, 6, 12, 50] - Cyanuric acid: less than 60 ppm if outdoors with complete restriction indoors [3] - Low water turbidity [6] |
| **Water filtration and turnover (n=3)** |
| - Ensure the pool water turnover and replenishment is achieved [23, 47] at least once daily [23] - Observe a usage interval of at least 30 seconds for water slides that are operated with untreated water [44] |
| **Cleaning (n=10)** |
| - Keep swimming pools properly cleaned [22] - Clean or brush the pool tiles and walls once a week (or more if needed) to reduce slime and biofilm [10, 16, 23] - Store cleaning products out of pool water or perimeter overflow systems [42], and out of reach of children [1, 15, 27, 31] - Ensure products are appropriately stocked to account for additional start up usage and possible delays in stock delivery [2] |
| **Quality control (n=23)** |
| - Monitoring (n= 14)   - Monitor pool chemistry, cleaning and disinfection throughout the day [6, 9, 15, 23, 27, 47, 48], at least twice per day and hourly when in heavy use [6, 7], or before opening and mid-afternoon [48]   - Monitor the free chlorine concentrations in bath water at all times [2], at least two times daily and more often if the pool is being used often [50]   - Test pH at least two times daily and more often if the pool is being used often [50]   - Conduct routine testing for safety of the water throughout the swimming season [49]   - Monitor filter pressure and backwash, as needed [42]   - Ensure optimal maintenance of water treatment devices [47]   - Maintain thorough monitoring records of disinfectant levels, pH measurements, cleaning and maintenance activities [6, 7, 48]   - Ensure appropriate inspections are carried out on circulation pumps, strainers, dosing pumps, injection points, water chemistry controllers, heaters and all other plantroom equipment for condition and confirm operation [23, 33], while minimizing the use of pool heaters [23]   - Employees of competent and authorized laboratories e.g. Institute of Public Health should monitor the water quality [36]   - Conduct a full inspection by Certified Pool Operator of all systems and water quality [42]   - Testing for COVID-19 in recreational waters at swimming areas is not recommended [49] |
| - Compliance with rules and regulations (n=18)   - Ensure the pool chemistry and other water quality parameters are maintained in accordance with State or local rules and regulations [5, 6, 15, 16, 23, 27, 28, 33, 37, 42, 45, 46, 48], CDC considerations [10, 21, 27], WHO strategies [47], licensing conditions [9], and/or manufacturers’ guidelines [34]   - Ensure the plant room is functioning based on the site-specific operations manual [33] |
| - Planning (n=1)   - Set a plan for the management of ordinary situations and for emergencies i.e., accidental releases of feces or biological fluids in pool water [47] |

## **Managing frequently touched surfaces (n=48)**

| **Cleaning and disinfecting areas (n=43)** |
| --- |
| 1. Toilets/restrooms/dressing rooms (n= 36)  - Faucets, sinks, soap, paper towel dispensers, hand dryers, toilet flush controls; surfaces of restrooms [1, 3, 5, 7, 13, 14, 15, 17, 20, 23, 31, 33, 34, 37, 42, 43, 49, 50] - Supplies for handwashing (including soap, disposable cleaning tools, materials for drying hands) [1, 4, 5, 8, 11, 15, 30, 32, 33, 34, 39, 50] - Baby changing stations [1, 3, 5, 15, 31] - Showers [5, 8, 9, 11, 15, 17, 18, 22, 25, 39, 40, 45, 47, 49, 50] - Dressing or changing rooms and lockers [7, 11, 12, 13, 17, 18, 20, 21, 29, 33, 34, 36, 37, 39, 40, 42, 43, 45, 47] |
| 1. Pool and beach area (n=28)  - Pool decks [3, 10, 18, 21] - Beach equipment (e.g., chairs, umbrella, canoes, kayak, rowboats, pool noodles, kickboards, and balls) [1, 5, 11, 15, 32, 36, 49, 50] - Tables, deck chairs, lounge chairs, sunbeds, floating equipment and benches, kickboards [1, 3, 4, 5, 7, 10, 14, 15, 17, 19, 20, 21, 23, 26, 27, 28, 29, 30, 32, 33, 36, 42, 45, 48, 49] |
| 1. Common areas/surfaces (n=34)  - Furnishings, entrance hall, waiting rooms, service areas, break rooms and common areas [4, 5, 13, 15, 19, 31, 37, 39, 40, 42, 47, 50] - Door knobs/handles [1, 3, 5, 7, 10, 11, 13, 15, 17, 18, 21, 23, 26, 30, 31, 32, 33, 37, 38, 39, 40, 42, 49, 50] - Phones [3, 14, 23, 42] - Light switches/lock boxes [1, 3, 7, 17, 23, 31, 32, 42] - Drinking/water fountains [1, 3, 7, 8, 10, 11, 14, 15, 23, 31, 36, 42, 48] - Vending machines [3, 15] - Any emergency shut off controls (typically found on spas) [3] - Stair railings, handrails, slides, structures for climbing, and pool ladders or lifts [1, 3, 5, 7, 8, 10, 11, 14, 15, 19, 20, 21, 22, 23, 26, 29, 31, 33, 39, 40, 42, 49, 50] - Entrances and exits [49] - Parent seating areas [49] - Payment devices [49] - Water bottle refill station [49] |
| **Frequency of cleaning and disinfection (n=33)** |
| - Before opening and after closing [4, 6, 18, 42] - Hourly [12, 18, 20, 33, 36] - Every 2 hours [7, 17, 29, 39] - Every 4 hours [9] - Once, twice, three time or more daily [1, 4, 5, 6, 8, 10, 11, 13, 14, 15, 16, 29, 40, 42, 49, 50] - Cleaning shared objects between uses [1, 5, 7, 8, 10, 11, 12, 13, 14, 15, 17, 19, 20, 21, 22, 24, 27, 29, 33, 34, 37, 38, 40, 42, 44, 49] |
| **Handling of towels (n=10)** |
| - Forbid sharing personal items such as towels; towels and consumables are to be changed after each use [9] - Consider requiring guests to provide their own towels [19, 25, 32, 37], and equipment [25, 37] - If provided;   - Store towels in covered, sanitized containers that are clearly delineated clean versus soiled [17, 32]   - Use appropriate temperatures when washing and drying towels to ensure sanitation (hot water for washing, ensuring towels are completely dried) [1, 15, 19, 17, 21, 26]; wash according to the manufacturer’s instructions [1, 19] |
| **Additional control measures (n=38)** |
| - Establish a schedule to document cleaning and disinfection [1, 6, 7, 11, 17, 23, 29, 42, 49] and post it in a visible location [1] - Establish a cleaning protocol [11, 23] or cleaning checklist [49] that includes defined areas and equipment that need to be cleaned [3, 23] - Discourage/prohibit from sharing objects that are difficult to clean, sanitize, or disinfect or that are meant to come in contact with the face (e.g. goggles, nose clips, and snorkels or caps) [1, 8, 10, 13, 14, 15, 16, 19, 20, 21, 23, 25, 27, 32, 33, 43, 44, 49, 50], from sharing utensils or other common objects [30] or consider a withholding period that allows any virus particles on these surfaces to die [38] - Restrict use of facilities for which disinfection between different users is not practical (e.g. single shower cubicles, changing rooms, shared furniture, shared personal floating devices) [4, 5, 8, 10, 14, 15, 17, 18, 19, 21, 23, 29] - Provide no-touch appliances or foot pedal installations e.g., at paper towel dispensers, soap dispensers, garbage bins, trash cans and at drinking fountain [1, 8, 11, 17, 21, 42, 43, 49, 50]; non-automatic entrance doors remain open [33, 40] - Reception/contactless transactions/online transaction/counter tops, keypads [5, 11, 17, 18, 21, 26, 27, 29, 30, 33, 34, 35, 39, 40, 42, 49] - Label containers for used and clean equipment [1, 15] - Follow federal guidelines (CDC, EPA) on what specific products shall be used and how; use products that meet EPA’s criteria for use against SARS-CoV-2 and that are appropriate for the surface [1, 3, 7, 10, 15, 17, 21 29, 31, 32, 42, 49] or local guidelines [5, 8, 20, 25] - Train staff about sanitation protocols [4, 5, 6] and on how to clean and use cleaning products safely [10, 22, 30, 49] - Close the place for 24 hours, 72 hours, or 120 hours to deactivate viruses [5, 18, 29] - Suspend the use of balls and any toys/games normally used for communal play (volleyball, water basketball, etc.), other shared exercise equipment [3, 26, 33], wristbands and handstamps [15] - Require patrons to bring their own trash bags, drink, and supplies (i.e. carry in/carry out rule) [49] - Have cleaning products available for patrons to self-clean shared surfaces before and after use [29], such as showers [15] - Do not use chlorinated pool water as a surface disinfectant solution as it is inadequate [3, 31, 49] - Separate containers for used equipment that have not been cleaned and disinfected from those of cleaned equipment [50]; separate furniture that needs to be cleaned and disinfected [50] |

## **Ventilation of indoor spaces (n=21)**

| - Ensure that ventilation systems of indoor spaces operate properly [1, 5, 12, 15, 21, 26, 27, 33, 44, 50] and meet current standards for ventilation and dehumidification [16] - Increase circulation of outdoor air [5, 26, 27, 29, 47, 49, 50] by opening windows [1, 3, 5, 12, 15, 16, 20, 26, 32, 33, 50] and doors [1, 5, 12, 15, 16, 33, 50], using fans [1, 5, 10, 15, 16, 20, 50], via the building management system [33], or other methods [1, 5, 15, 16, 50]; as long as it does not pose a risk to swimmers [5, 10, 12, 15, 16, 29] - Ensure restroom exhaust fans are functional and operating at full capacity when the building is occupied [1] - Promote best achievable air exchange and renewal, trying to consider exchange flows from the outside and avoiding internal circulations or ventilation from an indoor environment to another [47] - Increase frequency of air filter replacement and heat, ventilation and air conditioning (HVAC) cleaning for indoor pools and aquatic centers [1, 3, 17, 23, 31] - When scheduling swimming events, consider running a purge sequence starting 3 hours before an event and turn the system back to normal ventilation 1 hour before the event to allow environmental stabilization, if the air handling system has a purge mode. Run the purge mode again for 2 hours after the event [1] - Hang the air handling system’s time clock to introduce code ventilation 24 hours per day (no off cycle) [1] - “For facilities where a central ventilation system is not used, window air conditioning units or unit ventilators should be adjusted to maximize fresh air intake into the system, blower fans should be set on low speed and pointed away from room occupants to the extent possible. Ceiling fans should be adjusted so that fins are rotating in a direction that draws air up toward the ceiling rather than down onto occupants. Window fans should be turned to exhaust air out of the window in the direction of the outdoors. Window fans that blow air into a room or free-standing fans that only serve to circulate existing air around a room should not be used.” [49] - Consider using ultraviolet germicidal irradiation (UVGI) as a supplement to help inactivate SARS-CoV-2, especially if options for increasing the delivery of clean air are limited [1] |
| --- |

## **Screening and management of sickness (n=45)**

| **Screening for COVID-19 symptoms and precautions (n=37)** |
| --- |
| Pool users, swimmers, and patrons (n=26)   - Screen for symptoms [8, 9, 17, 19, 20, 21, 28, 30, 31, 32, 40, 47] - Do temperature checks [45, 47, 50], - Ask about contact with confirmed cases [28] - Complete screening electronically, or else, ensure regular cleaning of used material [11, 18] - Increase contact tracing [11, 17, 20,] (e.g., via encouraging customers to download Care19 Diary and Care19 Exposure apps [27], or having attendance record/log [5, 10, 22, 29, 33, 38, 40, 42] that is physical or electronic [34] such as the use of QR codes [38], application (COCOA) [37] - Facilitate tracking of the spectators by pre-online registration of passes for practice and tickets for competitions, and manual registrations at the entrance [50] |
| Employees and trainers (n=27)   - Screen for symptoms [1, 5, 6, 7, 11, 17, 18, 19, 21, 23, 26, 28, 30, 32, 33, 41, 42, 47, 48, 49] - Do temperature checks [1, 11, 17, 20, 21, 32, 37, 45, 47, 50] - Ask about contact with confirmed cases [11, 28, 32] - Self-monitor [5, 11, 15, 17, 21, 25, 26, 41, 49] - Train employees on symptom awareness [1, 11, 19, 24, 26] - Have screening protocol [5, 23] and maintain screening log [7, 11, 18, 33] - Maintain a log of employees on premise over time, to support contact tracing [49] |
| **Symptomatic and high-risk individuals (n=38)** |
| Pool users, swimmers, and patrons (n=31)   - Individuals with symptoms, suspected or confirmed infection, or contact with suspected or confirmed case:   - Inform employee [1, 43], health care provider [1, 8, 15, 31, 34, 40, 50], and state public health [20, 34]   - Prohibit swimming [7, 11, 14, 18, 34, 41]   - Isolate [1, 5, 8, 11, 14, 15, 20, 21, 26, 31, 33, 34, 40, 41]   - Assess risk, disinfect, and assist in contact tracing [34]   - Stay at home [1, 7, 8, 13, 14, 17, 18, 19, 20, 25, 28, 31, 34, 40, 43, 46, 47, 50]   - Educate on symptoms, when to stay home, and when to safely end home isolation [1, 7] - High risk pool users, swimmers, and patrons (n=13)   - Designate specific time to access without general population [5, 8, 21, 26]   - Take extra precautions [27, 29, 30]   - Avoid going to pool [19, 23, 31, 43, 48, 49] |
| Employees and trainers (n=35)   - Individuals with symptoms, and having been in contact with suspected or confirmed COVID-19 case:   - Inform employer [1, 5,11, 21, 26, 39, 49] and health care provider (for testing, tracking and/or provision of care) [1, 5, 6, 11, 20, 29, 33, 49] or health officials (e.g., COVID-19 Public Hotline) [1, 11, 15, 17, 21, 31, 33, 34] and exclude from work [48] or leave immediately [33]   - Prohibit swimming [11, 14, 18, 34, 41]   - Isolate [1, 5, 8, 11, 14, 15, 20, 21, 26, 31, 33, 40, 41]   - Assess risk, disinfect, and assist in contact tracing [34]   - Take necessary subsequent measures [35] and wear mask wear masks until leaving the facility [11, 26]   - Stay at home [5, 6, 10, 11, 13, 14, 16, 17, 18, 19, 20, 21, 23, 25, 26, 28, 29, 30, 31, 33, 34, 35, 37, 41, 42, 43, 46, 47, 48, 49]   - Return to work after 2 [43] or 3 asymptomatic days [18, 28], or after 3 asymptomatic days and 10 days have passed since first symptoms [15, 26, 30], or after 14 days [11]; return after 10 days since symptom onset AND 24 hours improvement in symptoms AND fever-free [29]   - Return to work after 10 days [26] or 14 days [11] have passed since first positive diagnostic test for employees who do not develop symptoms - Educate on when to stay home and when to safely end home isolation [1] - High risk employees and trainers (n=5)   - Reassign work or allow tele-work to minimize contact with others [10, 15, 16, 26, 28] |
| **Policies and procedures (n=26)** |
| - Use signs to notify individuals of the business’s COVID-19 mitigation plan and indicate that any person with COVID-19 symptoms may not enter the premises [18, 19, 24, 26, 27, 31, 33, 48, 49] - Use signs to notify individuals of measures taken in case of symptomatic customers [19, 36] or employees [19] - Establish policies on how to manage sickness [1, 18, 21, 29, 30, 43] including prompt identification and isolation of sick individuals [1, 15, 26, 27, 29, 43]; contacting a health provider [1, 15, 29, 41, 43]; planning for facility closure for cleaning and disinfection [1, 11, 17, 15, 21, 26, 33, 34, 42]; and maintaining confidentiality of cases [1, 11, 21, 28, 29, 33] - Monitor absenteeism [1, 10, 16] and have a sick leave policy that is flexible and non-punitive [1, 15, 17, 23, 26, 28, 41, 42] or paid [26, 29] - Have expanded family/medical leave for specific reasons (self-quarantining, testing) [26, 29]; adhere to federal guidance pertaining to paid leave [49] - Have return-to-work guidance in accordance with CDC criteria to discontinue home isolation [1, 18] - Have a plan to ensure required staffing [10, 16, 28] - Avoid retaliating against workers for raising concerns about COVID-19 related safety and health condition [49] - Wait 24 hours or as long as practical before beginning cleaning and disinfection when a person has COVID-19 symptoms or tests positive is detected onsite [1] - Develop a plan for arranging transportation for the person sick with COVID-19 [1], provide a dedicated bathroom for the sick person to use if possible, and make sure others do not use it until it can be properly cleaned and disinfected [1] - Designate a COVID-19 Point of Contact staff member to be responsible for responding to COVID-19 concerns. All staff should know who this person is and how to contact them [1, 15] - Designate first aid and trained medical officers who are able to triage and refer suspected cases for COVID-19 testing [50] - Appoint an employee safety team or point of contact to identify safety concerns [17] - Subject all athletes resuming to sport to standard protocol for COVID-19 testing [50] |

## **Delivering first aid (n=21)**

| **Providing first aid (n=10)** |
| --- |
| - Treat any victim as COVID-19 positive until otherwise determined [23, 42] - Provide and use one-way valve masks for CPR [10, 16, 43], provide pocket masks [26] or bag valve mask [23, 42] to eliminate mouth to mouth contact in case resuscitation is needed - Provide first aid training [10,], - At the end of the CPR, the rescuer must wash hands thoroughly with soap and water or alcohol-based gel; wash clothes as soon as possible, and make contact with the health authorities for further suggestions, if appropriate [4] - First aid rooms or facilities shall be stocked at all times, and cleaned and disinfected in accordance with Department of Health State Sanitary Code requirements and COVID-19 guidance [11] - Revise CPR protocol regarding contact with victim [23, 33, 39, 42] - Evaluate COVID-19 impact on rescue protocol in water incidents and on land incidents [23, 42] |
| **Lifeguard duties (n=12)** |
| - Lifeguard is not expected to   - Monitor hand washing [1, 5, 15]   - Monitor mask wearing [1, 3, 5, 15]   - Monitor social distancing [1, 3, 5, 15, 31, 42, 49]   - Perform other duties/duties that distract from the responsibilities of lifeguarding [23, 27]   - Perform “monitoring and cleaning” [29, 42] - Apart from providing adequate life guards in compliance with the licensing condition, consider deploying additional staff to patrol in the swimming pool area and remind swimmers to maintain social distancing [9] - Lifeguards must be trained to enforce beach social distancing and crowd control [11]; ~~will encourage physical distancing of 6 feet [48]~~ |
| **Lifeguard PPE (n= 10)** |
| - Lifeguards should wear PPE [4] - Provide each lifeguard with PPE [11, 31, 42], including a mask, gloves, hand sanitizer, CPR mask, safety glasses, and a surgical mask for beach goers who are pulled from the water [11] - Ensure adequate supply and reliable source of PPE [23, 33] - Lifeguards should wear face coverings   - When out of the water [5, 16]When social distancing cannot be maintained [3, 5, 11]; surgical-style mask, cloth mask, or other face covering when they are entering, exiting, or moving around work or common areas where contact with others cannot be avoided: this includes first aid, lost child searches, assisting handicapped individuals, and any other close contact with visitors [49]   - Except when performing water rescues or other lifesaving or emergency response activities [11, 49]   - Not necessary when lifeguard is sitting in an elevated chair overseeing the swim area [49] or when ensuring 6 feet social distance while on the lifeguard platform [3]   - Even if 6 feet social distance is maintained [31] - Lifeguards should use a type of face cloth that is easily removed before entering the water [3] - Lifeguards who cannot comply with face masks should be transferred to other duties [3] |
| **Lifeguard distancing and contact (n=8)** |
| - Mark off a 6-foot perimeter around lifeguard chairs when deck space allows [3, 26]; delineate exclusion line using cones or lines [49]; visitors, friends, and off duty lifeguards should not be within 6 feet of lifeguard stands [49] - Social distancing between lifeguards   - Limit the number of lifeguards at a given time on any stand or tower [11]   - Lifeguards should avoid sitting next to each other on lifeguard stands [49]   - Maintain social distance between lifeguards while providing the same coverage and protection necessary to ensure public safety, e.g. by adding lifeguard towers as necessary [1149] - Lifeguards should limit any close contact with other people to emergency situations [5] - Lifeguards and water safety personnel should abide by social distancing, except in the case of an emergency; i.e. rescuing a distressed swimmer, providing first aid, or performing cardiopulmonary resuscitation with or without an automated external defibrillator [1, 5, 11, 14, 19] |

## **Raising awareness (n=36)**

| **Channels and content (n=36)** |
| --- |
| - Signs (n=30)   - Social distancing [1, 3, 5, 6, 7, 8, 9, 10, 11, 13, 14, 15, 17, 20, 22, 26, 27, 29, 32, 40, 42, 43, 44, 48, 49, 50]; prohibiting congregations [1, 8, 17, 42, 49]   - Phone number to call the pool operator if violations are observed [49]   - Measures taken in case of symptomatic customers [1, 3, 5, 6, 7, 8, 10, 11, 15, 17, 18, 19, 21, 24, 26, 27, 31, 33, 36, 48, 49] or employees [19, 49] (e.g. no entry)   - Hand hygiene [1, 3, 5, 7, 8, 10, 15, 17,18, 20, 23, 27, 30, 42, 49, 50]; advising location of handwashing and sanitization stations [23, 42]; sanitization measures [11]   - Cough and sneeze etiquette [1, 5, 7, 10, 17, 20,49] and refraining from touching face, eyes nose with unwashed hands [1, 17, 49]   - Everyday protective measures [1, 50]   - Wear of cloth face covering [1, 3, 5, 9, 11, 17, 42, 50], personal protection protocols (face masks, gloves) [49]   - Refraining from wearing masks while in the water or entering water slide [3, 49]   - Capacity in pools [11, 20, 23, 26, 28, 31, 33, 42, 44], in restrooms [28]   - Traffic flow [33]   - Identification of COVID-19 symptoms [23, 32, 42, 48], reporting symptoms [11]   - Cleaning and disinfection protocols [11, 42, 49]   - Advising high risk individuals (i.e. more than 65 years, underlying health conditions) to refrain from entering [48]   - Advising of expectations and guidance [19]   - Avoiding hand shaking or physical contact [5]   - How to stop spread of COVID-19 [1, 29, 30, 32, 33, 36, 50]   - Acknowledging the risk of retracting COVID-19 when entering the facility [3], even under controlled conditions [42]   - Requiring full compliance with staff instructions [42]   - Showering before entering the pool [10]   - Avoiding standing, sitting, or otherwise blocking walkways or any identified narrow passage area [17, 49] - Broadcasts (n=7)   - Control of transmission: messages about how to stop spread [1, 15, 36, 50]; posted regularly on public address systems [1]   - Social distancing [5, 29, 49] - Other (e.g. emails, facility website, social media platforms, direct messaging to members, messages on entrance tickets) (n=7)   - ‘Awareness messages’ [50]   - Capacity limits, disinfection protocol, prohibiting entry in case of COVID symptoms [1, 5, 8, 15, 17, 42] |
| **Location of posted signs (n=18)** |
| - Entrance of facilities [1, 5, 15, 18, 19, 21, 22, 24, 26, 31, 33, 36, 40, 42, 44, 48] - Pool area [36, 42, 44] - Washrooms [1] - Cabin [44] - Locker room door [24] - Cloakroom area [40] - Kiosk and retail areas [33] - Highly visible locations [1, 15, 26, 30, 31] - Slide/diving platforms [44] - Spectator stand [9] |
| **Target audience considerations (n=2)** |
| - Signs should be in understandable language [8, 20] - Signs should be culturally appropriate [8] - Signs should take into consideration individuals’ age, ability and literacy level [8] |
| **Other measures (n=8)** |
| - Provide employees with information on help lines to access information or other support in reference to COVID-19 [26] - Make information available to workers about COVID-19 prevention and mitigation strategies, using methods like videos, webinars, or printed materials like FAQs [17, 26]; implement an awareness program on the preventive measures of COVID-19 [50] |

## **Vaccination (n=3)**

| - Even after a person has recovered from COVID-19 or is fully vaccinated, they should still continue to practice social distancing, wear a well-fitted mask, perform hand hygiene, and follow other precautions [23] - Facilities should be on the side of assuming that people entering their indoor site are unvaccinated [20] - In counties with one-week disease incidence rates over 35 per 100,000, masks must be worn in public indoor settings where ≥10 unvaccinated individuals or individuals of unknown vaccination status are present [20] - Staff should consider getting annual flu vaccination [34] |
| --- |

Abbreviations: CDC, Centers for Disease Control and Prevention; EPA, Environmental Protection Agency; WHO, World Health Organization

**References**

1. Barcala-Furelos R, Szpilman D, Abelairas-Gómez C, Alonso-Calvete A, Domínguez-Graña M, Martínez-Isasi S, et al. Plastic blanket drowning kit: A protection barrier to immediate resuscitation at the beach in the Covid-19 era. A pilot study. The American journal of emergency medicine. 2020;38(11).

2. Brian F. Ocean Shores businesses prepare for busy weekend amid pandemic. 2020.

3. Gerardo C, Sushma D, Raquel B, Kenji M. Harnessing testing strategies and public health measures to avert COVID-19 outbreaks during ocean cruises. medRxiv. 2021.

4. Marianne C. Signs, beaches and bodies in pandemic times. Media International Australia. 2021;178(1).

5. Patrícia dos Santos V, Roberto Miranda Ramos C. A COVID-19 E O DISTANCIAMENTO SOCIAL: quando a onda da internet substituiu a onda do mar para a prática de exercícios físicos. 2020;25(51).

6. Maria Emília Martins da Silva G, Eduardo Augusto Werneck R. A COVID-19 e sua Influência no Comportamento e Fruição das Praias Marítimas Urbanas de Balneário Camboriú, Santa Catarina, Brasil. Revista Turismo em Análise. 2020;31(3).

7. Efstratiou MA, Tzoraki O. Coronavirus survival on beach sand: Sun vs COVID-19. Marine pollution bulletin. 2021;167:112270.

8. Disjuncture as Well-Being in Youth Swimming: The Effects of the COVID-19 Pandemic on Everyday Associations and Routines. 2020;12(2).

9. Jennifer H. Risk perceptions of health and safety in cruising. 2020;6(4).

10. 정규선, 전정숙, 김기동. COVID-19의 장기화에 따른 심리상태가 해양관광지 선택과 방문의도에 미친 영향 - 강릉관광거점도시를 중심으로. 2020;19(5).

11. Brendan K, Chris ABZ, Thomas RA, George M, Nathan HA. Is it safer at the beach? Spatial and temporal analyses of beachgoer behaviors during the COVID-19 pandemic. Ocean & Coastal Management. 2021;205.

12. Liikkanen LA, Laukkanen JA. Sauna bathing frequency in Finland and the impact of COVID-19. Complementary therapies in medicine. 2021;56:102594.

13. Madeline F. Coronavirus infects Delaware teens who participated in senior week activities, others urged to get tested. 2020.

14. Montagna MT, De Giglio O, Calia C, Pousis C, Apollonio F, Campanale C, et al. First detection of severe acute respiratory syndrome coronavirus 2 on the surfaces of tourist-recreational facilities in Italy. Int J Environ Res Public Health. 2021;18(6):1-6.

15. Pereira LCC, de Sousa Felix RC, Brito Dias AB, Pessoa RMC, da Silva BRP, da Costa Baldez CA, et al. Beachgoer perceptions on health regulations of COVID-19 in two popular beaches on the Brazilian Amazon. Ocean & coastal management. 2021:105576.

16. Ashley LQ, Phi Yen N, Haley S, Samsung L, MacIntyre CR. Cruise ship travel and the spread of COVID-19 - Australia as a case study. international journal of travel medicine and global health. 2020;9(1).

17. Ramya G, Guru CS, Banodhe GK, Dominic D, Sharma HB. Resumption to swimming post COVID 19 lockdown. Journal of Clinical and Diagnostic Research. 2021;15(1).

18. Romano Spica V, Gallè F, Baldelli G, Valeriani F, Di Rosa E, Liguori G, et al. Swimming Pool safety and prevention at the time of Covid-19: a consensus document from GSMS-SItI. Annali di igiene : medicina preventiva e di comunita. 2020;32(5):439-48.

19. Romano-Bertrand S, Aho Glele LS, Grandbastien B, Lepelletier D, French Society for Hospital H. Preventing SARS-CoV-2 transmission in rehabilitation pools and therapeutic water environments. The Journal of hospital infection. 2020;105(4):625-7.

20. Sarah L, Bruce M. When the Beaches Close: Impact of COVID-19 upon County Fiscal Health in Florida. SSRN. 2020.

21. Vandana R. Jersey Shore comeback? Murphy says NJ beaches will reopen before Memorial Day as coronavirus cases decline. 2020.

22. Wroclaw Medical U. COVID-19 in Polish Ice Swimmers. clinicaltrialsgov. 2020.

23. Yang B, Li W, Wang J, Tian Z, Cheng X, Zhang Y, et al. Estimation of the potential spread risk of COVID-19: Occurrence assessment along the Yangtze, Han, and Fu River basins in Hubei, China. Sci Total Environ. 2020;746:141353.

24. Yoel. Analysis of the Impact of COVID-19 Sector on Parangtritis Beach Parawisata, Bantul District, Yogyakarta. SSRN. 2021.

25. It's still OK to take a beach day. Medical Marketing & Media. 2020;55(8):10-1.

1. The table does not include the one duplicate publication excluded [↑](#footnote-ref-1)
2. These are guidance documents; we included Ramya G et al, 2020 and Romano SV et al, 2020 in the review of strategies [↑](#footnote-ref-2)
3. “There are no more restrictions for a training group, however there are hardly any training groups of over 30 athletes in swimming” [↑](#footnote-ref-3)
4. The aerosols formed by the waves and the wind represent a natural source of airborne particles, which could transport the virus [↑](#footnote-ref-4)
